# The public health implications of the Paris Agreement: a modelling study

**DOI:** 10.1016/S2542-5196(20)30249-7

**Published:** 2021-02-10

**Authors:** Ian Hamilton, Harry Kennard, Alice McGushin, Lena Höglund-Isaksson, Gregor Kiesewetter, Melissa Lott, James Milner, Pallav Purohit, Peter Rafaj, Rohit Sharma, Marco Springmann, James Woodcock, Nick Watts

**Affiliations:** a UCL Energy Institute, University College London, London, UK; b Institute for Global Health, University College London, London, UK; c Air Quality and Greenhouse Gases Programme, International Institute for Applied Systems Analysis, Laxenburg, Austria; d Center on Global Energy Policy, Columbia University, New York, NY, USA; e Centre on Climate Change and Planetary Health, London School of Hygiene & Tropical Medicine, London, UK; f Department of Public Health, Environments and Society, London School of Hygiene & Tropical Medicine, London, UK; g Centre for Diet and Activity Research, MRC Epidemiology Unit, School of Clinical Medicine, University of Cambridge, Cambridge, UK; h Oxford Martin Programme on the Future of Food, Oxford Martin School, University of Oxford, Oxford, UK; i Centre on Population Approaches for Non-Communicable Disease Prevention, Nuffield Department of Population Health, University of Oxford, Oxford, UK

## Abstract

**Background:**

nationally determined contributions (NDCs) serve to meet the goals of the Paris Agreement of staying “well below 2°C”, which could also yield substantial health co-benefits in the process. However, existing NDC commitments are inadequate to achieve this goal. Placing health as a key focus of the NDCs could present an opportunity to increase ambition and realise health co-benefits. We modelled scenarios to analyse the health co-benefits of NDCs for the year 2040 for nine representative countries (ie, Brazil, China, Germany, India, Indonesia, Nigeria, South Africa, the UK, and the USA) that were selected for their contribution to global greenhouse gas emissions and their global or regional influence.

**Methods:**

Modelling the energy, food and agriculture, and transport sectors, and mortality related to risk factors of air pollution, diet, and physical activity, we analysed the health co-benefits of existing NDCs and related policies (ie, the current pathways scenario) for 2040 in nine countries around the world. We compared these health co-benefits with two alternative scenarios, one consistent with the goal of the Paris Agreement and the Sustainable Development Goals (ie, the sustainable pathways scenario), and one in line with the sustainable pathways scenario, but also placing health as a central focus of the policies (ie, the health in all climate policies scenario).

**Findings:**

Compared with the current pathways scenario, the sustainable pathways scenario resulted in an annual reduction of 1·18 million air pollution-related deaths, 5·86 million diet-related deaths, and 1·15 million deaths due to physical inactivity, across the nine countries, by 2040. Adopting the more ambitious health in all climate policies scenario would result in a further reduction of 462 000 annual deaths attributable to air pollution, 572 000 annual deaths attributable to diet, and 943 000 annual deaths attributable to physical inactivity. These benefits were attributable to the mitigation of direct greenhouse gas emissions and the commensurate actions that reduce exposure to harmful pollutants, as well as improved diets and safe physical activity.

**Interpretation:**

A greater consideration of health in the NDCs and climate change mitigation policies has the potential to yield considerable health benefits as well as achieve the “well below 2°C” commitment across a range of regional and economic contexts.

**Funding:**

This work was in part funded through an unrestricted grant from the Wellcome Trust (award number 209734/Z/17/Z) and supported by an Engineering and Physical Sciences Research Council grant (grant number EP/R035288/1).

## Introduction

To avoid the worst health effects of climate change, global annual greenhouse gas (GHG) emissions must halve by 2030 and reach net zero by 2050.^1^ This reduction cannot be achieved without strong and early GHG emission mitigation policies across every sector, particularly from fossil fuel use, which, across sectors, contributes 73% of total global GHG emissions.^2^

In the 2015 UN Framework Convention on Climate Change (UNFCCC) Paris Agreement, 196 states committed to reducing global average temperature rise to “well below 2°C above pre-industrial levels”.^3^ Alongside this target, countries announced nationally determined contributions (NDCs), which represent their sovereign efforts “to reduce national emissions and adapt to the impacts of climate change”.^2^ However, as they stood at the end of 2020, the NDCs were inadequate in their ambition, risking a global temperature rise of greater than 3°C by the end of this century.^4^

In addition to preventing the worst effects of climate change, efforts to reduce GHG emissions yield substantial near-term health benefits.^5^, ^6^ Well designed mitigation policies across the energy, built environment, food and agriculture, and transport sectors could result in cleaner air, improved housing, increased physical activity, and healthier diets.^7^, ^8^, ^9^, ^10^, ^11^, ^12^, ^13^, ^14^ These health benefits often confer economic benefits in the form of reduced health-care costs and a more productive workforce, which, in many instances, can outweigh the initial cost of the policy.^15^ The *Lancet* Countdown^16^ brings together more than 35 institutions from across the world to better understand the emerging health profile of a changing climate. In addition to its global monitoring system, the report aims to provide decision making support for national policy makers, and to highlight the health co-benefits of the implementation of the Paris Agreement.

**Research in context**

**Evidence before this study**

A 2018 review identified 36 studies that modelled the ancillary health effects of climate change mitigation in the areas of energy, transport, food and agriculture, household energy, and industry. Several studies have modelled the nationally determined contributions (NDCs) and mitigation in the energy sector and agriculture sector, consistent with the Paris Agreement, and have described the ancillary health effects that are associated with mortality due to ambient PM_2·5_ exposure as well as the effects of ozone on crop yields. Further studies have modelled the global ancillary health effects of dietary change consistent with the Paris Agreement, including specific policies such as taxes on red meat and processed meat. Studies quantifying the health effects of shifts from car transport to increased walking and cycling have mostly been at the city-level or state-level, although there have been studies for England and Wales. We searched PubMed for studies published in English from the database inception until April 9, 2020, using the search terms (“climate change” OR “greenhouse gas” OR “GHG”) AND “mitigation” AND (“co-benefit” OR “benefit” OR “health” OR “air pollution” OR “diet” OR “physical activity”), as well as the reference lists of and the studies citing the studies identified.

**Added value of this study**

This study is one of three articles from the *Lancet* Countdown that explores the engagement of health in policy making to achieve the goals of the Paris Agreement (ie, net-zero emissions). To our knowledge, this is the first study to present combined potential change in national-level ancillary health effects that are associated with dietary risk factors, physical activity, and ambient PM_2·5_ exposure resulting from mitigation policies in the food and agriculture, transport, and energy sectors, consistent with the Paris Agreement and Sustainable Development Goals, compared with national policies and existing commitments. We analyse Brazil, China, Germany, India, Indonesia, Nigeria, South Africa, the UK, and the USA in our study. We also show the potential gains of more health focused mitigation policies. To our knowledge, this study is also the first study to present national estimates of current and potential future health effects of walking and cycling for comparison across the selected countries.

**Implications of all the available evidence**

The results of this study contribute to the growing evidence of the health co-benefits of climate change mitigation—changes that affect the health of populations more rapidly than the health effects of climate change. This study also indicates the additional health benefits that could be achieved if health in all climate policies were adopted. A key role of researchers is to place greater emphasis on designing studies that contribute to decision making. The evidence presented here can be used to inform policy makers as they revise their NDCs to the Paris Agreement and the policies required to meet these targets. It will also add to the evidence base for the upcoming Intergovernmental Panel on Climate Change sixth assessment report. This study highlights the potential synergies that could be realised through addressing the COVID-19 crisis with a so-called green and healthy fiscal stimulus.

Panel **Scenarios used for climate and health modelling**

Baseline* **(2015)**

• Energy system: International Energy Agency (IEA) data for current fuels and energy system information was provided by the World Energy Outlook^20^
• Air pollution: using data from the IEA, pollutant (ie, PM_2·5_, sulfur dioxide [SO_2_], and nitrogen oxide [NOx]) emissions from different fossil fuels consumed by each sector were used to estimate the annual average particulate matter ambient air pollution concentrations using the Greenhouse gas—Air pollution Iteractions and Synergies (GAINS) system;^21^ other non-fossil fuel pollutants were estimated on the basis of sector activities and related assumptions on the technology adopted and population wealth^22^
• Greenhouse gas emissions: direct CO_2_ emissions from fossil fuels were estimated from the IEA fuel data and CO_2_ equivalent (CO_2_e) emissions were estimated from the precursor emissions (ie, SO_2_, NOx, fluorinated gases, and hydrochlorofluorocarbons [HCFCs]) based on the GAINS assumption for sectoral activities
• Diet: Data for present day diets were drawn from UN Food and Agriculture consumption data and were used to derive a national consumed diet
• Active travel: current activity levels for walking and cycling were derived from available travel and activity survey data for each country (or large city if no national survey was available)

**Current pathways scenario**† **(estimating for the year 2040)**

• Energy system: the IEA stated policies scenario (STEPS)^23^ was used to describe existing policy frameworks and ambitions that were relevant for the energy sector and accounted for current nationally determined contributions (NDCs)
• Air pollution: pollutant emissions for each sector for 2040 were derived from the IEA STEPS data for fuels and related sector activities to estimate ambient air pollution concentrations
• Greenhouse gas emissions: CO_2_ emissions from fossil fuels were estimated from the IEA STEPS data and CO_2_e emissions were estimated from the precursor emissions (ie, SO_2_, NOx, chlorofluorocarbons, and HCFCs) on the basis of the GAINS assumption for sectoral activities
• Diet: national diets and food system trends were projected using present-day business-as-usual baseline estimates for technological adoption, levels of food loss and waste, and overall dietary intake
• Active travel: minimal shift from current levels of physical activity

**Sustainable pathways scenario**‡ **(estimating for the year 2040)**

• Energy system: the IEA sustainable development scenario (SDS) describes the fuels and energy system features that are aligned with the Paris Agreement and sustainable development goal (SDG) 7 (affordable and clean energy); the SDS projected a global temperature rise of below 1·8°C with a 66% probability without reliance on global net-negative CO_2_ emissions; this scenario is equivalent to limiting the temperature rise to 1·65°C with a 50% probability; global CO_2_ emissions fall from 33 billion tonnes in 2018 to less than 10 billion tonnes by 2050 and are on track to net zero emissions by 2070
• Air pollution: pollutant emissions for each sector for 2040 were derived from the IEA SDS data for fuels and related sector activities to estimate ambient air pollution concentrations
• Greenhouse gas emissions: CO_2_ emissions from fossil fuels were estimated from the IEA SDS data along with related CO_2_e
• Diet: business-as-usual projections for technological progress, halving of food loss and waste, and dietary changes towards flexitarian diets
• Active travel: a net change in walking and cycling that is half of that achieved for the health in all climate policies scenario for each country

**Health in all climate policies scenario**§ **(estimating for the year 2040)**

• Energy system: IEA SDS was used for fuels and energy system features
• Air pollution: additional pollutant emission controls were added on top of those in the IEA SDS to further reduce ambient air pollution in the industrial and agriculture sectors
• Greenhouse gas emissions: CO_2_ emissions from fossil fuels were estimated from the IEA SDS data along with related CO_2_e
• Diet: increased ambitions of technological adoption, reduced food loss and waste by three quarters, and dietary changes to a combination of flexitarian diets (50%) and vegan diets (50%)
• Active travel: 75% of the population within each country walk or cycle over the course of a week

In the build-up to the UNFCCC's 26th Conference of the Parties—five negotiating cycles since the Paris Agreement—countries are reviewing their targets as part of a built-in so-called ratcheting up mechanism. This period of review is taking place in the context of a global pandemic, and short-term COVID-19 recovery packages will have an important role in contextualising longer-term climate change commitments.

In this study we aimed to show the possible GHG and population health effects resulting from current NDC targets within the energy, food and agriculture, and transport sectors, and the potential effects of more ambitious interventions consistent with the Paris Agreement. Further, we investigated the ancillary effects if countries were to more explicitly address health in the NDCs and place health in all climate policies. In this Article, scenarios were modelled for a set of nine representative countries that were selected for their contribution to global GHG emissions, and their global and or regional influence.

## Methods

### Overview

The countries selected for our study were: Brazil, China, Germany, India, Indonesia, Nigeria, South Africa, the UK, and the USA. These countries represent places across the world whose current and future development trajectories provide an interesting comparison of the potential health effects of the range of mitigation measures considered necessary to achieve the Paris Agreement and the unique challenges being addressed by the Sustainable Development Goals (SDGs).

### Selected countries' NDCs

A review of each country's NDC was undertaken as it related to the energy, food and agriculture, and transport sectors to evaluate their ambition for climate mitigation and the interventions they are taking to achieve their stated targets. Each of the selected countries submitted their first NDC in 2015, which outlined the respective actions they have proposed (appendix pp 1–2). As of December, 2020, of the countries included in our analysis, Germany and the UK have submitted updated NDCs (ie, the EU First NDC [updated submission] and the UK First NDC). China had also committed to carbon neutrality by the year 2060 but had not submitted an updated NDC.

The selected countries NDCs have a highly varied stance on climate change mitigation commitments. According to Climate Action Tracker, which evaluated NDCs prior to any updates on the basis of their stated commitments, their alignment to meet the Paris Agreement, and whether they constitute a fair contribution to global emission reductions, for some countries (eg, China and the USA) the proposed interventions remain highly misaligned to the Paris Agreement, while Germany's proposal to achieve a 55% reduction by 2030 is inadequate for an economy of its size.^17^ Some countries continue to make commitments, such as the UK, who have committed to reach net-zero carbon emissions by 2050. Overall, however, of the selected countries, only the contribution of India was “2°C compatible”.^17^ According to the Climate Action Tracker, the USA's efforts have been “critically insufficient”, those of China, Indonesia, and South Africa were “highly insufficient”, those of Brazil and the EU (including the UK) were “insufficient”, and Nigeria was not rated on its commitments.^17^

### Models and health outcomes

For the purposes of this study, three scenarios were developed to represent a range of possible future levels of ambition. The models that were used in this study track GHG emissions and air pollution,^18^ diets and diet-related health effects,^19^ and travel patterns and related health outcomes.^14^ All health outcomes are given as deaths avoided relative to the current pathways scenario (CPS) (panel).

#### Energy, GHG emissions, and air pollution

The International Energy Agency (IEA) world energy model provided estimates for fuels use in the year 2040.^26^ GHG emissions from energy, transport, and agriculture sectors for the years 2015 and 2040, as well as estimates for exposure to ambient PM_2·5_ and attributable premature mortality based on the fuels used, were calculated using the Greenhouse gas—Air pollution Interactions and Synergies (GAINS) model, which combines emissions calculations with atmospheric chemistry, dispersion coefficients, and environmental sensitivities.^18^ Details of world energy model, GAINS model, and health impact calculations are further described in the appendix (pp 3–7).

#### Food and agriculture

Changes in diet, technology, and food waste and associated diet-related health effects were estimated using an established food-system model, which is designed to model shifts consistent with the Paris Agreement and SDG 2 (zero hunger).^19^, ^27^ The effects of dietary change on chronic disease mortality were estimated using a comparative risk assessment framework consisting of nine risk factors and five disease endpoints (appendix pp 8–12).

#### Transport and physical activity

Baseline active travel mode share (ie, walking and cycling) was estimated for each country on the basis of survey data (appendix pp 13–14).^28^, ^29^, ^30^, ^31^, ^32^, ^33^, ^34^, ^35^ Using these data, we assessed the marginal metabolically equivalent task rate, relative risk reduction in disease risk, and reduction of mortality due to increased walking and cycling by age band for adults aged younger than 85 years.

#### Scenarios

Three scenarios were constructed to evaluate the potential greenhouse gas and health effects of the existing NDC commitments, as well as more ambitious pathways, for the selected countries. The scenarios (panel) were as follows: the countries' existing NDCs and stated national energy, air quality, transport, and health policies (ie, the CPS); a more ambitious scenario, aligned with the Paris Agreement and the SDGs (ie, the sustainable pathways scenario [SPS]); and a scenario that took steps to explicitly benefit health in climate change and related policies (ie, the health in all climate policies scenario [HPS]). For each country and sector, resource demands and their emissions were calculated for each scenario for the years 2015 and 2040, which is a mid point between the timelines for achieving the SDGs (ie, by the year 2030) and for achieving global net-zero GHG emissions (ie, by the year 2050). Deaths avoided were calculated for the year 2040 for each country and sector, comparing both the SPS and HPS with the CPS. Across the future scenarios, socioeconomic development patterns and population structures were aligned for the purpose of comparison.

### Role of the funding source

The funders of the study had no role in study design, data collection, data analysis, data interpretation, or writing of the report. All authors had full access to all the data in the study and had final responsibility for the decision to submit for publication.

## Results

**Figure 1:**
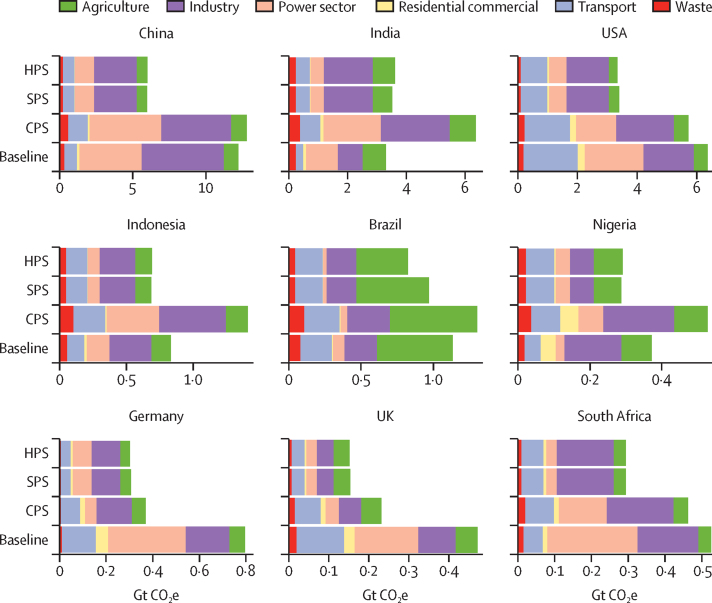
Total greenhouse gas emissions for select countries by sector for baseline (using data for the year 2015) and three scenarios in the year 2040 CPS=current pathways scenario. Gt CO_2_e=gigatonnes CO_2_ equivalent. HPS=health in all climate policies. SPS=sustainable pathways scenario.

The sectors and countries considered accounted for over 70% of global GHG emissions and 50% of the global population in 2015. China was the largest absolute emitter, followed by the USA (figure 1). Per capita, China's emissions were 9·1 tonnes compared with 16·1 tonnes for the USA (appendix p 18). Despite India's population being over four times that of the USA, its overall CO_2_ equivalent (CO_2_e) emissions were around 52% of the USA's. Nigeria had the youngest median age (17·9 years) and the lowest human development index (HDI) at 0·53, whereas Germany had the highest HDI (0·93) and the oldest median age (45·9 years; appendix p 17). High per capita CO_2_e emissions do not necessarily lead to a higher HDI: in 2015 the USA had the same level of development as the UK, but its per capita CO_2_e emissions were 2·8 times higher.

For the three countries with HDI coefficients above 0·9 (ie, Germany, the UK, and the USA), all show reductions in total primary energy and CO_2_e emissions in the three 2040 scenarios (figure 1). In the CPS, CO_2_e emissions show a substantial increase in all emerging economy countries, whereas under the SPS, which is designed to be compatible with the Paris Agreement, GHG emissions fall by between 20·6% (Nigeria) and 67% (UK), relative to existing levels, with the exception of India, where emissions rise by 6·2%. For the HPS, most countries follow a similar emissions trajectory to the SPS, although India and Brazil are more sensitive to changes in agriculture practices that affect emissions.

Under the SPS, PM_2·5_ concentrations (excluding natural sources) decrease for all countries and could be 73% lower on average in 2040 than for existing concentrations if the targets of the Paris Agreement and the Sustainable Development Goals are met. The largest reductions are seen in Nigeria (91% reduction), India (81% reduction), and Indonesia (79% reduction). These reductions are a combined effect of decarbonisation of economic activities, access to clean energy, as well the more ambitious emission controls.

The implementation of the HPS would offer further reductions in air pollution concentrations and show that countries with the highest projected concentrations under the CPS have the greatest reduction potential under the HPS. Air pollution concentrations in China decrease by 81% relative to the CPS, India's decrease by 86%, Indonesia's and Brazil's decrease by 88%, and Nigeria's decrease by 97%.

**Figure 2:**
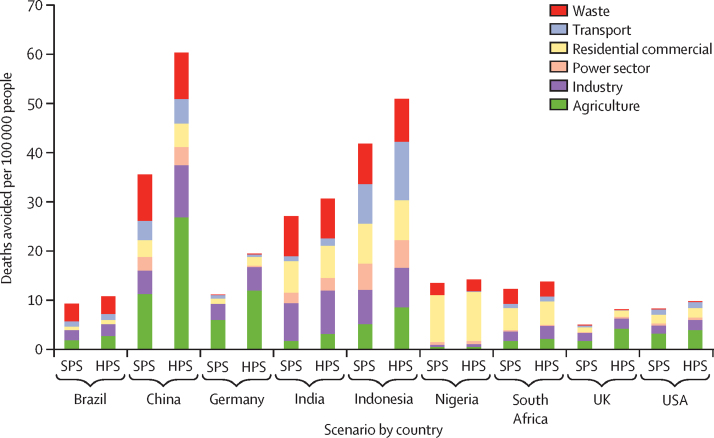
Number of deaths avoided attributable to PM_2·5_ concentration in the year 2040, relative to the CPS per 100 000 population, by sector, scenario, and country CPS=current pathways scenario. HPS=health in all climate policies. SPS=sustainable pathways scenario.

Across all countries considered, there would be 1·18 million deaths avoided in the year 2040 if the SPS were to be adopted instead of the CPS (table). Adjusted for each country's projected population size in 2040, the greatest effect is seen for Indonesia (42 fewer deaths per 100 000 population; figure 2). Strong reductions in deaths are also seen for China (36 per 100 000) and India (27 per 100 000). More moderate rates of deaths avoided are projected for South Africa (12 per 100 000), Nigeria (13 per 100 000), Germany (11 per 100 000), Brazil (9 per 100 000), the USA (8 per 100 000), and the UK (5 per 100 000).

**Table:** Deaths avoided in 2040 by scenario (relative to the current pathways scenario) and country (by absolute numbers of cases and per 100 000 population)

	**Daths avoided**			**Deaths avoided per 100 000 population**		
	Air pollution	Diet	Active travel	Air pollution	Diet	Active travel
**Brazil**						
SPS	21 069	328 040	56 224	9	143	24
HPS	24 456	336 270	102 386	11	147	45
**China**						
SPS	503 467	2 409 640	440 757	36	167	31
HPS	855 807	2 810 400	809 324	60	195	56
**Germany**						
SPS	8770	143 770	2856	11	188	4
HPS	15 614	143 710	5631	19	188	7
**India**						
SPS	433 549	1 741 860	364 948	27	111	23
HPS	491 756	1 869 300	670 230	31	119	43
**Indonesia**						
SPS	130 541	301 970	37 759	42	97	12
HPS	159 129	321 630	71 62	51	103	23
**Nigeria**						
SPS	43 839	88 490	29 376	13	25	8
HPS	46 915	91 550	55 094	14	26	16
**South Africa**						
SPS	8409	97 160	19 341	12	159	32
HPS	9457	98 900	35 011	14	162	57
**UK**						
SPS	3458	98 420	21 486	5	139	30
HPS	5771	100 100	38 441	8	141	54
**USA**						
SPS	30 560	654 580	172 618	8	171	45
HPS	36 371	664 050	300 419	10	173	78
**All countries analysed**						
SPS	1 183 662	5 863 930	1 145 365	26	130	25
HPS	1 645 276	6 435 910	2 088 298	37	143	46

HPS=health in all climate policies scenario. SPS=sustainable pathways scenario.

The HPS further reduces mortality across all countries by 370 000 deaths. Rates of air pollution-related deaths avoided rise to 60 per 100 000 population for China under the HPS, relative to the CPS. Other middle-income countries show clear benefits, with large reductions in Indonesia (51 per 100 000) and India (31 per 100 000). Germany, Brazil, South Africa, the USA, and the UK all show additional reductions in mortality when adopting the HPS, with deaths avoided for Germany rising to 19 per 100 000 population compared with data projected for the CPS.

The health benefits of mitigation in the food and agricultural sector are broadly seen as a result of a transition to more nutritious diets, in the form of increased consumption of fruit and vegetables, and reductions in the consumption of red meat and processed foods. The health and carbon benefits vary depending on a population's local context and health profile, with several countries consuming well over the daily recommended dietary intake of red meat and others consuming considerably less. Nonetheless, as low-income and middle-income countries continue to develop, it will be important to ensure that diets evolve and change in a way that maximises human health and wellbeing. This study provides one set of possible scenarios and recognises that a variety of different diets and interventions could be compatible with the Paris Agreement.

In the SPS, diets change towards calorie-balanced flexitarian diets that contain moderate amounts of animal source foods and high amounts of nutrition-sensitive plant-based foods. The reduction in intake of red meat varies by country, ranging from no reduction in India, to a 86–92% reduction in South Africa, the UK, Brazil, China, Germany, and the USA. At the same time, the intake of fruits and vegetables increases by 7% in China, over 14–34% in India, Nigeria, the USA, and the UK, to 50–55% in Indonesia, Brazil, and Germany.

**Figure 3:**
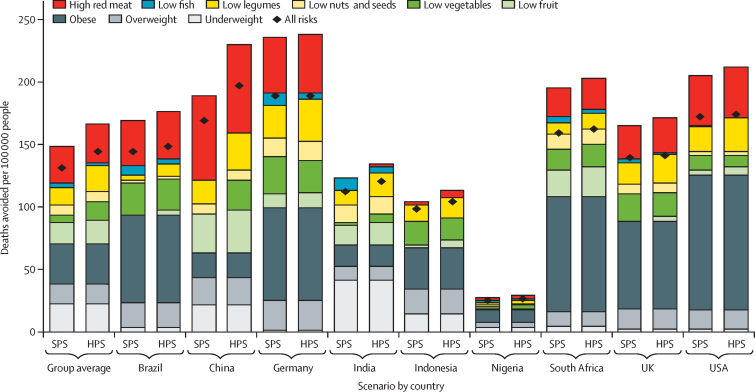
Number of deaths avoided attributable to dietary risks in the year 2040, relative to CPS per 100 000 population, by scenario and country The health impacts associated with the combination of all risks is smaller than the sum of individual risks because the former controls for co-exposure (ie, each death is attributed to only one risk factor). CPS=current pathways scenario. HPS=health in all climate policies. SPS=sustainable pathways scenario.

In the SPS, approximately 5·86 million deaths could be avoided in 2040 across the nine countries included in the analysis by switching from the CPS diet to a more plant-based, healthier diet containing less red meat^19^ (table). Half of the deaths avoided were due to changes in dietary risks, including decreased intake of red meat (22%), increased intake of fruits and vegetables (15%), legumes (9%), nuts and seeds (6%), and fish (3%), whereas the other half was due to reductions in obesity (22%), being underweight (15%), and being overweight (11%). Per population, Germany (188 per 100 000 population), the USA (171 per 100 000), and China (167 per 100 000) had the largest number of deaths avoided, followed by South Africa (159 per 100 000), Brazil (143 per 100 000), and the UK (139 per 100 000; figure 3). The fewest deaths avoided per 100 000 population were in India (111 per 100 000), Indonesia (97 per 100 000), and Nigeria (25 per 100 000).

In the HPS, diets become progressively more plant based, with half of populations adopting diets as per the SPS, and half adopting calorie-balanced vegan diets. Compared with the SPS, these dietary changes were associated with 572 000 additional deaths avoided (table). Across countries, the number of deaths avoided increased by 10%, ranging from 0 to 2% in Germany, the USA, the UK, and South Africa, over 3 to 7% in Nigeria, Indonesia, and India, to 17% in China (figure 3).

In the high-income countries included in this study, the degree to which active (ie, walking and cycling) travel increased under the SPS and the HPS varied as a function of the 2018 levels. In countries with low levels of car ownership, such as Nigeria, India, and South Africa, active travel is projected to continue in a downward trend in the CPS, but to be largely stabilised and maintained under the SPS and the HPS. For all countries, we recognise that high levels of voluntary participation in active travel are dependent on urban form, suitable infrastructure, and safety from traffic and other sources of danger.

**Figure 4:**
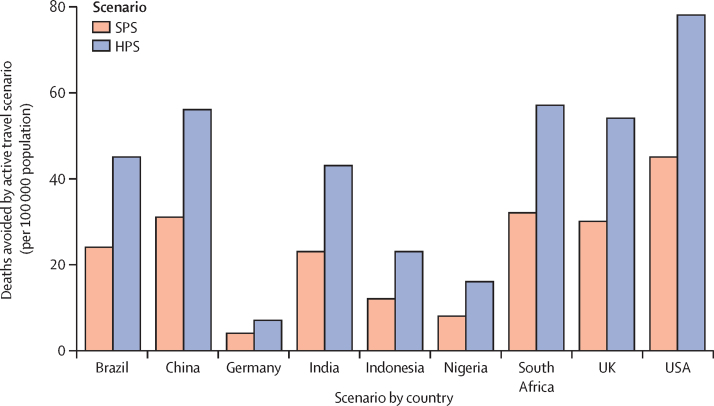
Number of deaths avoided in the year 2040 under the SPS and the HPS per 100 000 population, relative to the CPS CPS=current pathways scenario. HPS=health in all climate policies. SPS=sustainable pathways scenario.

As a result of the improved active travel participation rates, the total number of deaths avoided in the SPS, relative to the CPS in 2040, would be 1·15 million across the nine countries (table). The greatest gains are in the USA (45 deaths avoided per 100 000 population), South Africa (32 per 100 000), China (31 per 100 000), and the UK (30 per 100 000), with modest improvements for Indonesia (12 per 100 000), Nigeria (8 per 100 000), whereas Germany, which already has relatively high levels of active travel participation, shows modest improvements in deaths avoided (4 per 100 000; figure 4). Under the HPS, with greater participation and provided infrastructure, these overall trends in deaths avoided increase by 943 000 relative to the SPS.

## Discussion

Addressing climate change and achieving the Paris Agreement through strengthened NDC commitments to limit GHG emissions and the future risks of climate change will benefit health not only in the future but also in the present day. Our study showed that mitigation actions that reduce emissions and take commensurate actions on air pollution, improving diets, and active travel across a range of countries with different geographic and development contexts will offer substantial improvements to health. The longer governments wait to implement mitigation actions, the greater the delay in the number of deaths avoided.

The UN Emissions Gap report is unequivocal in its recommendation that countries collectively increase their commitments within the NDCs by three times as much as they currently are to limit temperature rise to “well below 2°C” as outlined in the Paris Agreement. Similarly, they recommend an increase by five times as much to reach a 1·5°C target.^4^

NDC commitments, as shown in the CPS, show the considerable impact on health of failing to improve climate ambitions to meet the Paris Agreement. By implementing the broader goals of the Paris Agreement and SDGs, health gains would be achieved through greater access and use of clean energy, reduced household and outdoor air pollution, improved diets with reduced waste, and increased participation in active travel. However, even greater gains could be made by placing health at the very centre of climate change mitigation and adaptation policies. The distinct advantage to aligning climate policies with health objectives is the greater political and societal buy-in for actions that have been seen purely in environmental terms thus far, further broadening their support by finding common ground among climate and health policy makers.

Although political, practical, institutional, and cultural barriers exist to realising the full extent of the HPS (as with the other scenarios), the main purpose of this analysis is to show why putting health at the forefront of the debate on climate change is crucial for protecting health. Poor quality air places a substantial burden on health, particularly among the most vulnerable communities around the world.^36^ Projected health effects from air pollution are strongly dependent on the implementation of national policies that drive reductions from fuel switching and also the application of highly feasible pollution controls that are commensurate with the level of effort required for the Paris Agreement and addressing the SDGs. By reducing pollution from electricity generation, household cooking, food and agriculture, industrial processes, and road transport, it is possible to reduce death and disease, particularly among women and children. This outcome is universal across the countries examined and is a key opportunity for policy action.

Achieving high rates of walking and cycling, as well as reducing car use, requires urban planning to provide sufficient population density and varied land use, considerate design to provide direct, safe, and high-quality walking and cycling routes, and accessible public transport. The risk of embedding sedentary lifestyles from travel practices should be avoided, while still recognising the complex nature of socioeconomic conditions and built environments across countries and cities.

Similarly, improving the health outcomes of diets requires that policy makers go far beyond food to addressing the cultural, economic, and behavioural factors that influence diets. The challenge of food quality and availability for different populations, along with the complex nature of food systems, presents a major barrier to improving diets. National diets hide the variation of calories being consumed among individuals, particularly in low-income settings, where a large number of people might have inadequate nutrition or low food availability. These challenges mean that large changes in food systems will have to happen to enable dietary changes at the population level.

There are several key limitations of this study. The modelling undertaken was conceptualised as a projection of the potential resources, emissions, and health effects of alternate future pathways. The uncertainties associated with modelling that uses complex and multifactorial methods are many and difficult to capture with standard estimates of confidence. In this Article, we evaluated the uncertainties through a qualitative approach that outlined key areas of uncertainty within the models and implications for the results, included as a table in the appendix (pp 15–16). Additionally, the interactions between changing dietary risks and changing physical activity levels were not modelled and thus their outcomes are not additive. Furthermore, the interaction between active travel and air pollution were not accounted for, but the benefits of active travel will increase as air pollution concentrations are reduced.^37^ Nevertheless, both of these interactions result in substantial health benefits, with enormous gains from behavioural interventions that are supported through policy and infrastructure within the transport and agriculture sectors.

For emerging economies, efforts to mitigate and adapt to climate change while realising the health benefits described in the Results, will require large financial support, including from the US$100 billion a year pledged by high-income parties to the UNFCCC.^38^ For all countries and regions, the necessary policy responses include a range of structural, technological, economic, and behavioural interventions across all social, cultural, economic, and political contexts.^5^, ^6^, ^39^ Leadership has been seen at the subnational level and synergy is required between top-down national commitments and bottom-up measures for communities to benefit from the full extent of these health co-benefits.^6^ An example of this type of synergistic action is the effort being undertaken to increase space for pedestrians to improve physical distancing and access to outdoor amenities, such as in Paris, Toronto, and Rome,^40^ which, in turn, supports physical and mental health.^41^ The key for instituting climate change and health actions beyond the present will be communicating the benefit of the adopted measures for the long term.

To achieve the Paris Agreement targets, annual global emissions must halve by 2030, reducing at an annual rate of 7·6%.^4^ In April, 2020, the daily emissions of some of the countries discussed in this Article decreased by a quarter during the height of COVID-19 lockdown measures, and early estimates suggest that emissions in 2020 could be 8% lower than in 2019, representing the largest ever year-on-year decrease.^42^, ^43^ However, these reductions do not reflect a decarbonisation of the economy: the underlying infrastructure in the energy system has not changed and emissions are expected to rise as economies recover from the COVID-19 lockdown.

In these recoveries, it is crucial that countries ensure that their recovery measures are consistent with the “well below 2°C” goal to ensure that one public health crisis is not replaced with another. The beginnings of these types of actions can be seen, with the UK's announced investment in walking and cycling initiatives, China's new infrastructure stimulus, and the EU's and South Korea's focus on Green New Deals as the cornerstones of their economic recovery post-COVID-19.^44^, ^45^, ^46^, ^47^ At the same time, some countries have strengthened their efforts since we did this analysis, with the UK and EU submitting stronger NDC targets, China announcing its commitment to achieving carbon neutrality before the year 2060, and the Joe Biden and Kamala Harris administration promising to commit to net zero emissions by the year 2050.^48^, ^49^, ^50^ But, even with these new announcements, the world is not yet on track to meet the goals of the Paris Agreement.^4^

Globally, and for each of the countries studied here, both the proposed and actual response to climate change up to now have been inadequate. This Article shows that this inadequate action creates a missed opportunity to improve the health of populations around the world today and in the future. Comparing the health effects seen in the CPS against a scenario that prioritises human wellbeing (ie, the HPS) makes this opportunity abundantly clear, with numbers of deaths avoided tallying in the millions by 2040.

The health and economic benefits from cleaner air, healthier diets, and more active communities are clear, and materialise across a range of development and societal trajectories. However, these interactions are not yet embraced in climate policies, with little reference to public health seen in current NDCs. The consideration of these co-benefits not only strengthens the case for further ambition to meet the climate change commitments stated in the Paris Agreement, but also creates opportunities for health professionals to work with policy makers, engineers, energy, transport and agriculture experts, and economists to ensure that human health is the foundation of all climate change policies. A HPS approach—placing health in the design, assessment, and implementation of policy responses to climate change—provides the opportunity to ratched ambition towards the goal of “well below 2°C” in a way that maximises good health and wellbeing.

## Footnotes

* The baseline scenario presents the current context for each country covering each of the sectors analysed for around the year 2015.

† Estimating for the year 2040, this scenario was designed to reflect stated policies in energy, air quality, transport, and food and agriculture sectors along with their NDC commitments on greenhouse gas emission reductions.

‡ Estimating for the year 2040, this scenario was designed to be aligned with the commitments of the Paris Agreement to limit the increase in global temperature to well below 2°C within the century and also to meet SDG2 (zero hunger), SDG3 (good health and wellbeing), SDG7 (affordable and clean energy), and SDG13 (climate action). The sustainable pathways scenario assumed the adoption of the best available abatement measures.

§ Estimating for the year 2040, the health in all climate policies approach seeks to systematically take into account the health implications of policy decisions, seeking synergies and avoiding harms to maximise population good health and wellbeing.^24^ This scenario includes measures that are in line with the Paris Agreement and SDGs but seeks to maximise ancillary health benefits. The health in all climate policies scenario assumes further adoption of pollution abatement measures under an accelerated rate.^25^
