# Correction to *Lancet Planet Health* 2021; 5: e74–83

**DOI:** 10.1016/S2542-5196(21)00083-8

**Published:** 2021-05-05

*Hamilton I, Kennard H, McGushin A, et al. The public health implications of the Paris Agreement: a modelling study.* Lancet Planet Health *2021; **5:** e74–83*—In this article there was an incorrect value given for the Indonesian health in all climate policies scenario in the table. Additionally, several minor typos have also been corrected. These corrections have been made as of May 5, 2021.

