# Supplementary material for: The public health implications of the Paris Agreement: a modelling study
Source: Lancet Planet Health. 2021 Feb 10;5(2):e74–83. doi: 10.1016/S2542-5196(20)30249-7 (PMC7887663; doi:10.1016/S2542-5196(20)30249-7)
Supplement: Supplementary appendix [file mmc1.pdf]

THE LANCET
Planetary Health

Supplementary appendix

This appendix formed part of the original submission and has been peer reviewed. We post it as supplied by the authors.

Supplement to: Hamilton I, Kennard H, McGushin A, et al. The public health implications of the Paris Agreement: a modelling study. *Lancet Planet Health* 2021; **5:** e74–83.

Responding to climate change for health: the public health implications of the Paris Agreement

Supplementary Appendix

Section 1: NDC Commitments and projections

Table S1. NDC for selected countries.[1,2]

Country / Sector	NDC key actions			
	**Overall**	**Energy**	**Agriculture and diet**	**Transport**
China	Projected 7%–15% emissions *increase* above 2015 levels by 2030. Peak emissions by 2030. 60 - 65% below 1990 carbon intensity of GDP.	Increase the share of non-fossil fuels in primary energy consumption to around 20% by 2030. Electricity to be 35% renewables by 2030. Industry target - Achieve effective control on emissions of HFC-23 by 2020	Emissions to be 'controlled'.<br><br>Afforestation to be 'vigorously enhance[d]'	Improved gasoline quality by 2020. Promote the walking and cycling in cities
Brazil	Projected 93% emissions increase above 1990 by 2030. Unconditional target 58% increase above 1990 (excluding LUC)	45% of renewables in the energy mix by 2030.	Increasing the share of sustainable biofuels to approximately 18% by 2030.<br><br>Restoring an additional 15 million hectares of degraded pasturelands by 2030 and enhancing 5 million hectares of integrated cropland-livestock-forestry systems (ICLFS) by 2030.	Promote efficiency measures
Germany	41% - 43% below 1990 level by 2030<br>55% emissions reduction target for 2030 from 1990 baseline	Proposed phasing out coal by 2038	Emit no more than 58-61 $MtCO_2e$/year by 2030	7-10 million electric vehicles by 2030
India	>50% increase in emissions by 2030. Unconditional target 33-35% below 2005 emissions intensity of GDP by 2030	Improve the efficiency of coal power plants. 40% of electricity to be non-fossil fuel by 2030.	"[The] long-term goal is to bring 33% of its geographical area under forest cover eventually".<br><br>Create a carbon sink of 2.5-3.0 $GTCO_2e$ through additional forest and tree cover by 2030.	Reducing emissions from transportation sector. Facilitate transition to electric vehicles.
Indonesia	Approximately 400% above 1990 levels Unconditional target 535% above 1990 levels	Renewable energy at least 23% of TPES in 2025 and at least 31% in 2050<br><br>Oil to be less than 25% of TPES in 2025 and less than 20% in 2050	N/A	N/A
Nigeria	20% unconditional (45% conditional) reduction below BAU. (not covered by CAT)	Work towards ending gas flaring by 2030<br><br>Work towards Off-grid solar PV of 13GW (13,000MW)<br><br>Improve efficiency	N/A	N/A

South Africa	Unconditional target 17%-78% above 1990. 33% to 39% increase above 1990 level.	Decarbonised electricity by 2050<br><br>23 Mt CO_2 CCS from the coal-to-liquid plant<br><br>Additional 15.8 GW for wind and 7.4 GW for solar by 2030<br><br>Decommission 35GW of coal by 2050	N/A	N/A
United States of America	Indicated intent to withdraw from Paris Agreement on November 5th 2020. Previous commitment 26%-28% reduction below its 2005 level in 2025<br><br>Emissions unchanged by 2030	Previous commitment to reduce emissions from the power sector by 32% below 2005 levels by 2030.	N/A	N/A
United Kingdom	UK Climate Change Act 2008 (2050 Target Amendment) Order 2019 requires target for at least a 100% net reduction of greenhouse gas emissions (compared to 1990 levels) by 2050	Coal phased out by 2025<br><br>Oil and gas over 70% of TPES in 2030	11% fall between 2019 and 2030.	Ban on petrol/diesel cars by 2035<br><br>Cycling/walking infrastructure upgrades
Key: colour indicates source	CAT projection	CAT NDC ratified excluding LULUCF	CAT policy description (usually post NDC)	NDC directly<br>N/A = no clear pledges

Section 2: Model methods

IEA World Energy Model (Energy and CO_2):

The International Energy Agency (IEA) maintain the World Energy Model (WEM),[3] which is a simulation model designed to replicate how energy markets function and is the main tool by which the IEA generate detailed sector-by-sector and regional projections for the World Energy Outlook scenarios.

The WEM is an energy supply, transformation and demand energy model that provide global estimates of the dynamics of the global energy system. The model relies on different end use sector models to estimate demand functions that are linked to the global energy balances supply and primary energy demand flows (see Figure S1).

The main assumptions of the model related to economic growth, demographics and technological developments. A range of dynamically linked sectoral models for electricity, oil and overall primary energy demand are interlinked with the latest country-level statistical inputs and observed market trends.

The output of the model is typically measured in the SI unit of Joules (e.g. Exajoules or Petajoules) or as an oil equivalent (i.e. Toe). Energy balances are produced and reported at a regional level. CO_2 emissions for those regions based on internationally agreed CO_2 factors.

The model outputs at the annual level over the scenario period, with historic data being updated to the latest available reported statistics, which typically include a 1 or 2 year lag.

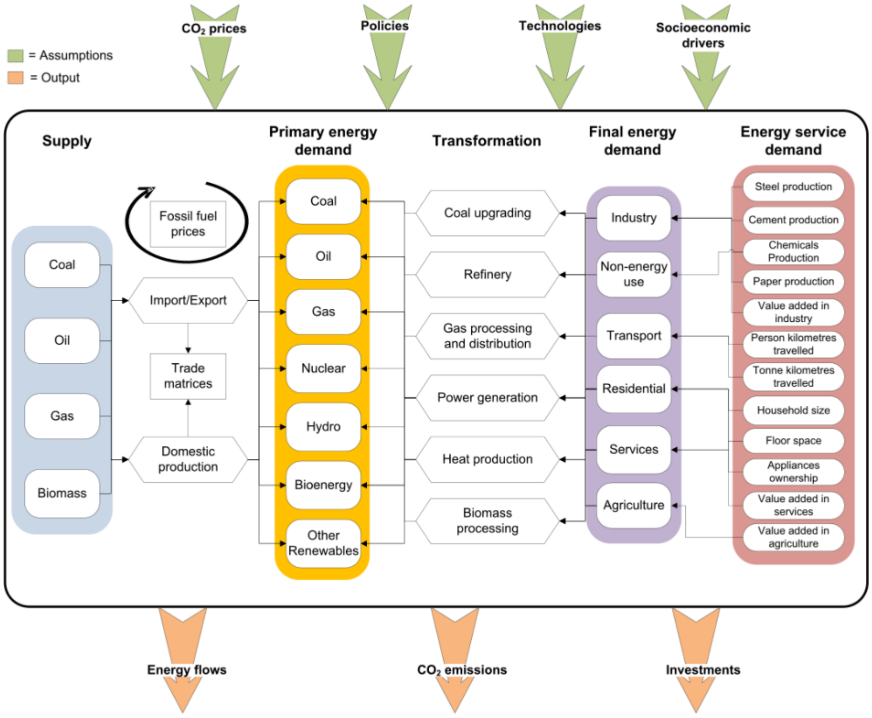

Figure S1. World Energy Model structure.[3]

GAINS

The GAINS (Greenhouse gas-Air pollution Interactions and Synergies) model explores cost-effective multi-pollutant emission control strategies that meet environmental objectives on air quality impacts (on human health and ecosystems) and greenhouse gases. GAINS, developed by the International Institute for Applied Systems Analysis (IIASA), brings together data on economic development, the structure, control potential and costs of emission sources, the formation and dispersion of pollutants in the atmosphere and an assessment of environmental impacts of pollution.

GAINS has been used to address air pollution impacts on human health from fine particulate matter and ground-level ozone, vegetation damage caused by ground-level ozone, the acidification of terrestrial and aquatic ecosystems and excess nitrogen deposition to soils, in addition to the mitigation of greenhouse gas emissions. GAINS describes the interrelations between these multiple effects and the pollutants (SO_2, NOx, PM, NMVOC, NH_3, CO_2, CH_4, N_2O, F-gases) that contribute to these effects at the regional scale.

The global version of the GAINS model which is used for this study employs a spatially disaggregated representation of the world in 180 source regions, which are either countries, provinces or sub-national aggregates. Among the countries considered in this study, China is represented at provincial level (35 provinces), India in 23 aggregates of states/union territories, the USA as mainland + Alaska, and all other countries as countries.

Activity projections are supplied by IEA's WEM in the WEM native region, sector and fuel disaggregation.[3] They are translated into the GAINS region, sector and fuel classification using the proportional downscaling algorithm reported by Rafaj et al (2013 and 2018).[4,5] The WEM model provides information on the future evolution of the energy system under various climate and energy policies for the following subsectors: power generation, fuel extraction and conversion, industry, transport and buildings. Not only combustion-related activities are modelled in WEM, also projections for industrial processes, e.g., iron and steel production, cement and aluminium manufacturing are developed. If some of the emission sources are not explicitly represented in WEM, they are derived from the socio-economic drivers such as population and economic growth, sectoral value added trends, etc. Examples of emitting sectors in GAINS not covered explicitly by WEM include livestock numbers, burning of agricultural residues, waste generation, brick production and other industrial process activities.

Energy consumption data from the WEM projections is distributed across the GAINS sub-regions (countries, states, provinces) based on shares derived from international and national energy and industrial statistics (see referenced examples).[6-9] The downscaling procedure also allocates energy consumption to detailed subsectors and fuel types in GAINS that are not explicitly provided by the energy model. These include various transport sub-categories, industrial demand activities split into furnaces/boilers as well as fuel conversion and processing.

For each of the source regions considered in GAINS, emission estimates for a particular emission control scenario consider (1) the detailed sectoral structure of the emission sources that emerges from the downscaling of the activity projection described above, (2) their technical features (e.g., fuel quality, plant types, etc.), and (3) applied emission controls (GAINS includes a database of over 1000 technical measures).

For each key source sector, the spatial patterns of PM and its precursors emissions are then estimated at a $0.5^o \times 0.5^o$ longitude–latitude resolution, based on relevant proxy variables (updated from Klimont et al 2017).[10] These estimates rely on the most recent updates of data on population distribution, road networks, plant locations, open biomass burning, etc. that were originally developed within the Global Energy Assessment project.[11] For the residential sector, a finer resolved emission distribution map has been developed at 0.1^o resolution, combining fine resolved gridded population with urban-rural classification, and estimates of prevalence of different fuel use in urban and rural areas.

CO_2

Computation of CO_2 emissions in GAINS follows the approach documented in Amann et al (2008) and is based on combining the exogenous activity data (e.g., the energy scenarios developed in WEM) and corresponding emission factors.[12] Removal efficiency of carbon capture and storage (CCS) installations are considered in the emission factors in power and industrial sectors. CO_2 emissions are computed with a bottom-up approach, for each economic sector in each GAINS region (subregion, country, province). Emission for historic years are

calibrated to the national GHG inventories reported by Parties to the UNFCCC, while also the emission factors are derived from the UNFCCC guidelines.[12]

Non-CO_2 greenhouse gases

The non-CO_2 greenhouse gases covered in the GAINS model framework are methane (CH_4), nitrous oxide (N_2O) and the fluorinated gases hydrofluorocarbons (HFCs), perfluorocarbons (PFCs) and sulphur hexaflouride (SF_6). Internally consistent analyses of technical mitigation potentials for global non-CO_2 greenhouse gases in the 2050 timeframe have been described in Höglund-Isaksson et al. (2020), Winiwarter et al. (2018), and Purohit and Höglund-Isaksson (2017).[13-15] The GAINS model relies on importing externally produced projections for activities in the energy and agricultural sectors, while projections for the generation of waste and wastewater and the use of F-gases in cooling and other applications are generated internally in GAINS in consistency with the macroeconomic projections of the energy scenario implemented. For this particular exercise, three alternative scenarios were developed for future non-CO_2 greenhouse gas emissions. All three use macroeconomic and energy sector activity specific drivers to 2040 that are consistent with the IEA World Energy Outlook 2018.[16] The current pathways scenario is developed in consistency with the energy projections of the associated New Policies Scenario (NPS), while an alternative low-emission scenario (sustainable pathways scenario) is developed in consistency with energy projections of the Sustainable Development Scenario (SDS) and assuming maximum implementation of existing technical mitigation potential for non-CO_2 greenhouse gases. A third emission scenario, the health in all climate policies scenario, builds on the latter scenario, but assumes in addition that widespread shifts towards more plant-based human diets take place. For the Baseline and low emission scenarios, projections of livestock numbers, fertilizer use, and crop area are developed in consistency with FAO long-term trends.[17] To reflect shifts in human diets, we use an alternative agricultural scenario developed by IIASA's GLOBIOM model for the 2019 report of the Food and Land Use Coalition.[18] This scenario simulates a shift towards an EAT Lancet diet under a constraint of global food security.

Assessment of fluorinated greenhouse gases (F-gases: hydrofluorocarbons (HFCs), perfluorocarbons (PFCs) and sulfur hexafluoride (SF_6)) emissions in the GAINS model follows the approach documented in Purohit and Höglund-Isaksson (2017) and Höglund-Isaksson (2017).[14,19] Activity data used to estimate HFC emissions in the years 2005, 2010 and 2015 is derived from HFC consumption reported by industrialized countries (Germany, UK and USA) to the UNFCCC. For developing countries, HCFC and HFC consumption data is extracted from available literature[14,20] and HFC inventories prepared by Climate and Clean Air Coalition (CCAC).[21] In addition, for each HFC emission source, the fraction of HCFC in the HFC/HCFC use is identified from reported baselines of parties to the Montreal Protocol and modelled in consistency with the phase-out schedule of HCFCs in the latest revision of the Montreal Protocol and including later baseline up-dates reported by the parties to the UNEP Ozone Secretariat and in the HCFC Phase-out Management Plans. For the development of the baseline scenarios in the timeframe to 2040, we use the existing model setup in GAINS, which for global scenarios uses drivers consistent with macroeconomic and energy sector projections from the IEA World Energy Outlook 2017.[22] Further details on model assumptions for estimating HFC, PFC and SF_6 emissions are provided in Purohit et al. (2017).[14]

Ambient $PM_{2.5}$ and health impact calculations

The general principle of ambient $PM_{2.5}$ calculations in GAINS has been discussed by Amann et al. (2011).[23] Owing to the history and evolution of the GAINS model over time, slightly different versions have been implemented in the European domain and in the global domain outside Europe. All versions rely on perturbation simulations of atmospheric chemistry transport models, in which emissions from a given source region and pollutant are reduced from base case, and the change in ambient concentration levels is used to calculate a linear transfer coefficient. Source pollutants considered for the formation of $PM_{2.5}$ are primary $PM_{2.5}$ (PPM), SO_2, NOx, NH_3, and VOC. For PPM, the transfer coefficients are split into one describing low-level emissions from residential combustion and traffic, and one for all other sources, to account for different atmospheric dispersion characteristics of emissions injected at different heights.

Ambient $PM_{2.5}$ calculations for Europe have been described by Kiesewetter et al.[24,25] Linear transfer coefficients were derived based on EMEP model simulations (5 met years 2006-10) from region-pollutant specific emissions to 0.5^o x 0.25^o grid, then downscaling of low-level PPM within the grid cell to a finer 0.125^o x 0.0625^o grid ("7km") and urban polygons inside the 7km grid, using a linear relationship between sub-grid PPM emission density and calculated $PM_{2.5}$ concentrations derived from a full-year simulation of the CHIMERE CTM.[26] Low-

level emissions considered for the downscaling include the domestic (SNAP 2), road transport (SNAP 7), and off-road transport (SNAP 8) sectors. Urban-rural split of emissions is done at the level of sub-7km grid, to redistribute the 7km emissions into the urban polygon and the rest of the grid cell. This sub-7km split is done by population density for SNAP 2 and 7 except heavy duty trucks.

Ambient $PM_{2.5}$ calculations outside Europe have been described by Amann et al. (2020).[27] They follow a very similar approach, however using slightly different resolution and CTM model versions. Also, they are more explicit in terms of differentiating urban and rural low-level emission sources. Base case and reduction simulations (15% reduction runs for pollutants PPM total, PPM low-level (SNAP 2+7), SO_2, NO_X, NH_3, VOC, with met year 2015) have been run with the EMEP CTM at 0.5° resolution, with either an Asia-wide domain as used in the UNEP-CCAC Assessment of Air Pollution in Asia and the Pacific, or a global domain for all other regions. On top of these ordinary transfer coefficient calculations, two global simulations with 0.1° resolution were conducted for the meteorological year 2015: a base case simulation, and a simulation in which all residential emissions from located urban areas (all pollutants) were reduced by 30% simultaneously. This additional reduction run was used to split the PPM low-level transfer coefficient into urban and non-urban, and to split the SO_2 and NO_x transfer coefficients into low-level urban and the rest, and to increase the resolution of ambient $PM_{2.5}$ calculations from all low-level sources to 0.1° globally outside Europe.

Deaths attributable to ambient $PM_{2.5}$ for regions other than Europe are calculated using the methodology of the WHO assessment on the burden of disease from ambient air pollution,[28] which relies on disease specific integrated exposure response relationships (IERs) developed within the Global Burden of Disease 2013 study and are presented in Figure S2.[29]

The population attributable fraction PAF_{dja} of air-pollution related deaths from disease d in region j and age a are calculated as

$$PAF_{dja} = \frac{\sum_i \frac{pop_{ji}}{pop_j}(RR_{dai} - 1)}{1 + \sum_i \frac{pop_{ji}}{pop_j}(RR_{dai} - 1)} \qquad (1)$$

where i represent the 0.1° grid cells hosting population pop_{ji} belonging to region j. RR_{dai} is the disease and (possibly) age specific relative risk as calculated from the integrated exposure response functions for $PM_{2.5}$ concentration levels in that spatial unit.

Deaths attributable to ambient $PM_{2.5}$ exposure are calculated by multiplying the PAF_{dja} from Eq. (1) with age specific baseline cases of deaths d_{dja} from disease d in region j:

$$pd_{dja} = PAF_{dja} \cdot d_{dja} \qquad (2)$$

Age-specific numbers of deaths from individual diseases are estimated from published numbers for the year 2010 in the Global Burden of Disease 2013 project, which were obtained from the GBD data query tool. Age-specific projected total deaths for each GAINS region are taken from the UN World Population Prospects 2017.[30] We assume that while total age-specific deaths vary according to the UN projections, the relative shares of individual diseases contributing to age-specific deaths remain unchanged in the future.

For Europe, calculations for deaths attributable to ambient $PM_{2.5}$ follow the WHO Europe methodology and apply exposure-response relationships for all-cause mortality among population over 30 years of age as reported under the REVIHAAP assessment.[31] Equations (1) and (2) are applied without further age differentiation to total deaths above 30 years of age, using the approximation

$$pd_j \approx \beta \cdot PM_j \cdot d_j$$

with $\beta = 0.00588$, PM population-weighted mean anthropogenic $PM_{2.5}$ and d_j the number of non-accidental deaths over 30 years of age in each country j.

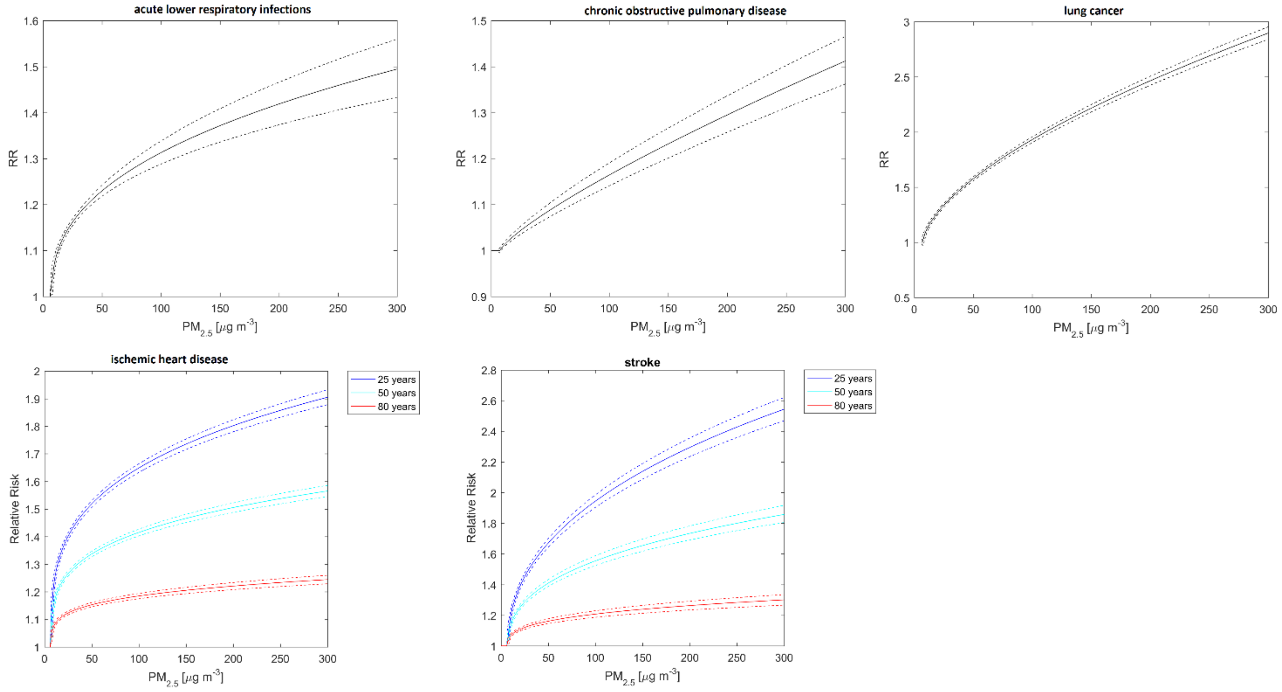

Figure S2. Disease-specific integrated exposure-response curves (mean and 95% confidence interval) for ambient $PM_{2.5}$ exposure.

Diet Model (Diet, Health):
The estimates of the diet-related health co-benefits were based on estimates by Springmann and colleagues.[32,33] The estimates differed by degree of technological progress, reduction in food loss and waste, and dietary change. For this analysis, we adopted the following scenario combinations:

- *Current pathways scenario*: business-as-usual projections for technological progress, food loss and waste, and dietary change
- *Sustainable pathways scenario*: business-as-usual projections for technological progress, halving of food loss and waste, and dietary changes towards flexitarian diets
- *Health in all climate policies scenario*: ambitious levels of technological progress, reducing food loss and waste by three quarters, and dietary changes to a combination of flexitarian diets (50%) and vegan diets (50%)

Diet projections and scenarios
We estimated baseline and projected food intake by adapting food demand projections from the International Model for Policy Analysis of Agricultural Commodities and Trade (IMPACT) that were based on a harmonised dataset of country-specific food availability data, and we adjusted those for food waste at the household level.[34,35] Future projections of food demand were income-dependent and followed a middle-of-the-road socio-economic development pathway (shared socio-economic pathway 2, SSP2), as developed by the climate change research community[36-38] and are in line with other projections.[39,40] For estimating the prevalence of underweight (BMI<18), overweight (25<BMI<30) and obesity (BMI>30) in each country, we fitted log-normal distributions to WHO estimates of mean BMI and the prevalence of overweight and obesity using a cross-entropy method that jointly minimised the deviation of the prevalence data, and we projected weight changes by using correlations between changes in mean BMI and changes in food availability.[41]

The flexitarian and vegan scenarios were based on recommendations of the EAT-Lancet Commission on Healthy Diets from Sustainable Food Systems. The flexitarian dietary patterns contain no processed meat, low amounts of red meat (including beef, lamb, pork) and sugar, moderate amounts of poultry, dairy and fish, and generous amounts of fruits, vegetables, legumes, and nuts. In the vegan dietary pattern, all animal source foods were replaced to one third by fruits and vegetables and to two thirds by legumes. The dietary patterns were regionalised for each country by preserving the current national preferences for types of grains, fruits, red meat and fish.

Health analysis
To analyse the implications of dietary change for chronic disease mortality, we constructed a comparative risk assessment framework nine risk factors and five disease endpoints.[42] The risk factors included high consumption of red meat, low consumption of fruits, vegetables, nuts and seeds, fish, and legumes, as well as being underweight (BMI<18.5), overweight (25<BMI<30), and obese (BMI>30). The disease endpoints included coronary heart disease (CHD), stroke, type-2 diabetes mellitus (T2DM), cancer (in aggregate and as colon and rectum cancers), and respiratory disease (which is associated with changes in weight).

We estimated the mortality and disease burden attributable to dietary and weight-related risk factors by calculating population impact fractions (PIFs) which represent the proportions of disease cases that would be avoided when the risk exposure was changed from a baseline situation to a counterfactual situation. For calculating PIFs, we used the general formula:[29,42,43]

$$PIF = \frac{\int RR(x)P(x)dx - \int RR(x)P'(x)dx}{\int RR(x)P(x)dx}$$

where $RR(x)$ is the relative risk of disease for risk factor level x, $P(x)$ is the number of people in the population with risk factor level x in the baseline scenario, and $P'(x)$ is the number of people in the population with risk factor level x in the counterfactual scenario. We assumed that changes in relative risks follow a dose-response relationship,[43] and that PIFs combine multiplicatively, i.e. $PIF = 1 - \prod_i(1 - PIF_i)$ where the i's denote independent risk factors.[43,44]

The number of avoided deaths due to the change in risk exposure of risk i, $\Delta deaths_i$, was calculated by multiplying the associated PIF by disease-specific death rates, *DR*, and by the number of people alive within a population, *P*:

$$\Delta deaths_i(r,a,d) = PIF_i(r,d) \cdot DR(r,a,d) \cdot P(r,a)$$

where PIFs are differentiated by region *r* and disease/cause of death *d*; the death rates are differentiated by region, age group a, and disease; the population groups are differentiated by region and age group; and the change in the number of deaths is differentiated by region, age group and disease.

We used publicly available data sources to parameterize the comparative risk analysis. Mortality data were adopted from the Global Burden of Disease project,[45] and projected forward by using data from the UN Population Division.[46] Baseline data on the weight distribution in each country were adopted from a pooled analysis of population-based measurements undertaken by the NCD Risk Factor Collaboration.[47]

The relative risk estimates that relate the risk factors to the disease endpoints were adopted from meta-analyses of prospective cohort studies.[48-54] In line with the meta-analyses, we included non-linear dose-response relationships for fruits and vegetables, nuts and seeds, and fish, and assumed linear dose-response relationships for the remaining risk factors. As our analysis was primarily focused on mortality from chronic diseases, we focused on adults aged 20 year or older, and we adjusted the relative-risk estimates for attenuation with age based on a pooled analysis of cohort studies focussed on metabolic risk factors,[55] in line with other assessments.[29,56]

Table S2 provides an overview of the relative-risk parameters used in the analysis. The selection of risk-disease associations used in the health analysis was supported by available criteria used to judge the certainty of evidence, such as the Bradford-Hill criteria used by the Nutrition and Chronic Diseases Expert Group (NutriCoDE),[56] the World-Cancer-Research-Fund criteria used by the Global Burden of Disease project,[57] as well as NutriGrade (Table S3).[58] The certainty of evidence supporting the associations of dietary risks and disease outcomes as used here were graded as moderate or high with NutriGrade,[51,53,59] and/or assessed as probable or convincing by the Nutrition and Chronic Diseases Expert Group,[56] and by the World Cancer Research Fund.[60]

For the different diet scenarios, we calculated uncertainty intervals associated with changes in mortality based on standard methods of error propagation and the confidence intervals of the relative risk parameters. For the error propagation, we approximated the error distribution of the relative risks by a normal distribution and used that side of deviations from the mean which was largest. This method leads to conservative and potentially larger uncertainty intervals as probabilistic methods, such as Monte Carlo sampling, but it has significant computational advantages, and is justified for the magnitude of errors dealt with here (<50%) (see e.g. IPCC Uncertainty Guidelines).

Caveats

In the comparative risk assessment, we used relative risk factors that are subject to the caveats common in nutritional epidemiology, including small effect sizes and potential measurement error of dietary exposure, such as over and underreporting and infrequent assessment.[61] For our calculations, we assumed that the risk-disease relationships describe causal associations, an assumption supported by the existence of statistically significant dose-response relationships in meta-analyses, the existence of plausible biological pathways, and supporting evidence from experiments, e.g. on intermediate risk factors.[48,49,51-54,56,59,62,63] However, residual confounding with unaccounted risk factors cannot be ruled out in epidemiological studies. Additional aspects rarely considered in meta-analyses are the importance of substitution between food groups that are associated with risks, and the time lag between dietary exposure and disease.

To address potential confounding, we omitted risk-disease associations that became non-significant in fully adjusted models, in particular those related to milk intake,[64,65] but potential confounding might also exists for the association between increased fish intake and reduced CHD risk.[66-69] The quality of evidence in meta-analyses that covered the same risk-disease associations as used here was graded with NutriGrade as moderate or high for all risk-disease pairs included in the analysis (SI Table 3).[51,53,59] In addition, the Nutrition and Chronic Diseases Expert Group and the World Cancer Research Fund graded the evidence for a causal association of ten of the 12 risk-disease associations included in the analysis as probable or convincing.[56,60] The relative health ranking of leading risk factors found in our analysis was similar to existing rankings that relied on different relative-risk parameters and exposure data.[57,70]

As exposure data, we used a proxy of food consumption that was derived from estimates of food availability that were adjusted for the amount of food wasted at the point of consumption.[34,71] An alternative would have been to rely on a set of consumption estimates that has been based on a variety of data sources, including dietary surveys, household budget and expenditure surveys, and food availability data.[72,73] However, neither the exact combination of these data sources, nor the estimation model used to derive the data have been made publicly available. For some individual countries, using dietary surveys would also have been an alternative. However, underreporting is a persistent problem in dietary survey,[74,75] and regional differences in survey methods would have meant that our results would not be comparable between countries. In contrast to dietary surveys, waste-adjusted food-availability estimates indicate levels of energy intake per region that reflect differences in the prevalence of overweight and obesity across regions.[47]

Table S2. Relative risk parameters (mean and low and high values of 95% confidence intervals) for dietary risks and weight-related risks.

Food group	Endpoint	Unit	RR mean	RR low	RR high	Reference
Red meat	CHD	100 g/d	1.15	1.08	1.23	Bechthold et al (2019)
	Stroke	100 g/d	1.12	1.06	1.17	Bechthold et al (2019)
	Colorectal cancer	100 g/d	1.12	1.06	1.19	Schwingshackl et al (2018)
	Type 2 diabetes	100 g/d	1.17	1.08	1.26	Schwingshackl et al (2017)
Fish	CHD	15 g/d	0.94	0.90	0.98	Zheng et al (2012)
Fruits	CHD	100 g/d	0.95	0.92	0.99	Aune et al (2017)
	Stroke	100 g/d	0.77	0.70	0.84	Aune et al (2017)
	Cancer	100 g/d	0.94	0.91	0.97	Aune et al (2017)
Vegetables	CHD	100 g/d	0.84	0.80	0.88	Aune et al (2017)
	Cancer	100 g/d	0.93	0.91	0.95	Aune et al (2017)
Legumes	CHD	57 g/d	0.86	0.78	0.94	Afshin et al (2014)
Nuts	CHD	28 g/d	0.71	0.63	0.80	Aune et al (2016)
Underweight	CHD	15<BMI<18.5	1.17	1.09	1.24	Global BMI Collab (2016)
	Stroke	15<BMI<18.5	1.37	1.23	1.53	Global BMI Collab (2016)
	Cancer	15<BMI<18.5	1.10	1.05	1.16	Global BMI Collab (2016)
	Respiratory disease	15<BMI<18.5	2.73	2.31	3.23	Global BMI Collab (2016)
Overweight	CHD	25<BMI<30	1.34	1.32	1.35	Global BMI Collab (2016)
	Stroke	25<BMI<30	1.11	1.09	1.14	Global BMI Collab (2016)
	Cancer	25<BMI<30	1.10	1.09	1.12	Global BMI Collab (2016)
	Respiratory disease	25<BMI<30	0.90	0.87	0.94	Global BMI Collab (2016)
	Type 2 diabetes	25<BMI<30	1.88	1.56	2.11	Prosp Studies Collab (2009)
Obesity (grade 1)	CHD	30<BMI<35	2.02	1.91	2.13	Global BMI Collab (2016)
	Stroke	30<BMI<35	1.46	1.39	1.54	Global BMI Collab (2016)

	Cancer	30<BMI<35	1.31	1.28	1.34	Global BMI Collab (2016)
	Respiratory disease	30<BMI<35	1.16	1.08	1.24	Global BMI Collab (2016)
	Type 2 diabetes	30<BMI<35	3.53	2.43	4.45	Prosp Studies Collab (2009)
Obesity (grade 2)	CHD	30<BMI<35	2.81	2.63	3.01	Global BMI Collab (2016)
	Stroke	30<BMI<35	2.11	1.93	2.30	Global BMI Collab (2016)
	Cancer	30<BMI<35	1.57	1.50	1.63	Global BMI Collab (2016)
	Respiratory disease	30<BMI<35	1.79	1.60	1.99	Global BMI Collab (2016)
	Type 2 diabetes	30<BMI<35	6.64	3.80	9.39	Prosp Studies Collab (2009)
Obesity (grade 3)	CHD	30<BMI<35	3.81	3.47	4.17	Global BMI Collab (2016)
	Stroke	30<BMI<35	2.33	2.05	2.65	Global BMI Collab (2016)
	Cancer	30<BMI<35	1.96	1.83	2.09	Global BMI Collab (2016)
	Respiratory disease	30<BMI<35	2.85	2.43	3.34	Global BMI Collab (2016)
	Type 2 diabetes	30<BMI<35	12.49	5.92	19.82	Prosp Studies Collab (2009)

Table S3. Overview of existing ratings on the certainty of evidence for a statistically significant association between a risk factor and a disease endpoint. The ratings include those of the Nutrition and Chronic Diseases Expert Group (NutriCoDE),[56] the World Cancer Research Fund,[60] and NutriGrade.[51,53,59] The ratings relate to the risk-disease associations in general, and not to the specific relative-risk factor used for those associations in this analysis.

Food group	Endpoint	Association	Certainty of evidence
Fruits	CHD	reduction	NutriCoDE: probable or convincing;<br>NutriGrade: moderate quality of meta-evidence
	Stroke	reduction	NutriCoDE: probable or convincing<br>NutriGrade: moderate quality of meta-evidence
	Cancer	reduction	WCRF: strong evidence (probable) for some cancers<br>NutriGrade: moderate quality of meta-evidence for colorectal cancer
Vegetables	CHD	reduction	NutriCoDE: probable or convincing<br>NutriGrade: moderate quality of meta-evidence
	Cancer	reduction	WCRF: strong evidence (probable) for non-starchy vegetables and some cancers<br>NutriGrade: moderate quality of meta-evidence for colorectal cancer
Legumes	CHD	reduction	NutriCoDE: probable or convincing<br>NutriGrade: moderate quality of meta-evidence
Nuts and seeds	CHD	reduction	NutriCoDE: probable or convincing<br>NutriGrade: moderate quality of meta-evidence
Fish	CHD	reduction	NutriCoDE: probable or convincing<br>NutriGrade: moderate quality of meta-evidence
Red meat	CHD	increase	NutriGrade: moderate quality of meta-evidence
	Stroke	increase	NutriGrade: moderate quality of meta-evidence
	Cancer	increase	WCRF: strong evidence (probable) for colorectal cancer<br>NutriGrade: moderate quality of meta-evidence for colorectal cancer
	Type-2 diabetes	increase	NutriCoDE: probable or convincing<br>NutriGrade: high quality of meta-evidence

NutriCoDE: Nutrition and Chronic Diseases Expert Group

NutriGrade: Grading of Recommendations Assessment, Development, and Evaluation (GRADE) tailored to nutrition research

WCRF: World Cancer Research Fund

Active Travel (Travel, Health):
We conducted a rapid review of active travel in nine countries (listed below) to assess the percentage of population who regularly walk and cycle, and to inform scenario development.

We reviewed recent data on active travel, extrapolated to a 2018 baseline and projected forward to 2040 under different scenarios.

The pre-baseline active travel data was taken from:

- The USA was based on the National Household Travel Survey data.[76]
- Germany was based on the Mobility in Germany.[77]
- The UK was based on the National Travel Survey data of England and Wales.[78]
- Brazil was based on São Paulo Metropolitan Region data (2012).[79,80]
- China was based on the Chinese Nutrition and Health Surveillance (2010–2012) data.[81]
- South Africa was based on the National Household Travel Survey (2013).[82]
- Indonesia was based on the Greater Jakarta data (2018).[83]
- India was based on Census 2011 data.[84]
- Nigeria was based on Lagos Metropolitan Area (2015).[85]

We assessed the Current Pathways Scenario (CPS) of active travel in 2040 and compared it against the two alternative scenarios:

- Health in all climate policies scenario (HPS): under this scenario it was assumed that in 2040, 75% of the population will be active (walk and/or cycle). This was assumed based on Germany's active travel pattern[77] – about 72% of population age between 70 and 79 do any cycling and/or at least 30 minutes of main-mode walking in a week. The mean walking duration was assumed as 210 minutes per pedestrian per week; and mean cycling duration was assumed as 180minutes per cyclists per week; this mean distance is assumed based on German data. [77]
- Sustainable pathways scenario (SPS): In 2040, the additional percentage of people who are active (walk and/or cycle) in this scenario will be half of the net change (HPS-CPS) in the HPS. The mean walking duration (210minutes) and cycling duration (180minutes) are assumed same as the HPS.

The HPS and SPS were assessed against the CPS, to calculate the net change in number of people who walk and/or cycle and associated health impacts in 2040 only.

The step-by-step approach/process which have been adopted for each country is discussed in the 'Lancet – Active Travel Appraisal Method' spreadsheet. Overall method is discussed in the next section.

Overall Method
Overall, we adopted a data hierarchy and calibrated other data set to that. The data hierarchy is (in descending order):

- Individual level trip data over a year.
- Individual level trip data over 1 week.
- Individual level trip data over 1 day
- Summary statistics from travel survey on mode share on 1 day
- Summary statistics on commuting only on 1 day

Traditionally, transport planning/modelling processes are largely used to assess the number of trips by mode and purpose, rather than number of people who are active. Thus, active travel mode share was more readily published than the number of people who are active.

The pre-baseline data on percentage of people who are active in India, Indonesia and South Africa was assessed by converting active travel commuting mode share in these countries based on an Indian medium sized city (Visakhapatnam) data.[86] Nigeria's mode share was converted to people who are active based on Accra data.[79] The number of people who are active in the UK, China, Germany and Brazil was available (per-baseline year).

Daily active travel pattern was converted to weekly as a week of active travel is more representative of typical/habitual physical activity e.g. person who doesn't travel 1 day but are active on other days of the week. Data on weekly active travel pattern was not available for most of the countries, expect for Germany and the UK.

Ratio of daily to weekly active pattern was assessed based on the UK active travel data. This ratio was applied to convert a day active travel data to a week – in the USA, Brazil, China, South Africa, Indonesia, India and Nigeria. Germany active travel data on a day to a week active pattern was not applied to other countries as the UK data was more representative/applicable to other countries than Germany.

Weekly active travel data was then converted to the pre-baseline year. This was then extrapolated to the 2018 baseline and 2040 CPS based on annual growth factors – it was assumed that there will be no change in percentage of people who are active in the USA, the UK, Germany and Brazil; whilst, based on available evidence a small annual decrease in active travel was assumed China, South Africa, India, Nigeria and Indonesia. In the HP scenario it was assumed that in 2040, 75% of the population will be active (walk and/or cycle). In the SP scenario it was assumed that in 2040, the additional percentage of people who are active will be half of the net change (HPS-CPS) in the HPS. The mean walking duration will be 210minutes and cycling duration be 180minutes) in both the HP and SP scenarios.

We calculated the percentage of people who 'walk', 'cycle' and 'walk or/and cycle' in each of the three scenarios (CPS; HPS; SPS) in 2040 and in CPS in 2018 by age band. The percentage of people active by age band was available for Germany, England and the USA; for other countries, an equal percentage of active population within each age band was assumed. This assumption likely overestimates the baseline and thus underestimates the potential gain.

Using the percentage of the population active, active travel duration, and standard Marginal Metabolically Equivalent Task (MMET) rates for walking and cycling, we calculated increases in population levels of physical activity. Using dose response relationships from Kelly et al. (2014),[87] we calculated the potential impact fraction for each age band. These data was used to calculate number of deaths avoided due to increases activity (walk and/or cycle) in the HPS and SDS scenarios as compared to the CPS in 2040.

Limitations and exclusions

Some of the key limitations and exclusions underpinning this active travel appraisal are as follows:

- There is considerable uncertainty about the possible impacts of the COVID-19 on future travel pattern, which are not considered as part of this work. In future, sensitivity tests can be undertaken when evidence is available on post COVID-19 travel pattern to show a higher/ lower active travel scenarios and associated health benefits.
- Impact of emerging technologies such as micromobility on active travel is not considered as part of this work.
- Limited active travel data is available for middle-income countries.
- Only 'main-mode' walking and/or cycling trips were considered.

Section 3: Uncertainties

Table S4. Uncertainties across the models and scenarios.

Sector and exposure pathway	Uncertainty component	Description
**All pathways**	Population and demographic projections for estimating exposure	We use the UN Department of Economic and Social Affairs World Population Prospects (2012 and 2017 Revisions)[30,46] data for population by age group in 2040. These estimates are based on all available sources of data on population size, levels of fertility, mortality and international migration. The greatest difference in the population projections for 2040 is for South Africa which has been revised from 61 million (2012) to 69 million (2017) and subsequently 70 million (2019). Across all countries considered 2017 projections for 2040 were 2% higher than 2012 projections. Probabilistic Projections including median, 80% and 95% prediction intervals are available here: https://population.un.org/wpp/Download/Probabilistic/Population/
**Energy and air pollution**	Policy uptake of the three scenarios (CPS, SPS, HPS)	The World Energy Model scenarios model the impact of specific policies and measures on energy demand, production, trade, investment needs, supply costs and emissions. The scenarios are based on the implementation of current commitments and plans (Stated Policies Scenario) and a least-cost implementation of policies for the achievement of SDG 7, SDG 3.9 and the Paris Agreement (Sustainable Development Scenario). The IEA modelling makes use of the RCP2.6. The policies database (https://www.iea.org/policies) is used to inform these scenarios. Governments will likely to continue to intervene in energy markets in the coming decades, but there is much uncertainty regarding what policies and measures will be introduced and the success of these.[3]
	Projections of energy demand	The IEA uses population and economic projections to drive the energy demand projections in the World Energy Model. The population assumptions do not differ between the scenarios. The population numbers within the World Energy Model scenarios are well aligned (within 2%) of the UN World Population Prospects.[30]
	Greenhouse gas emissions and exposure to air pollutants	The Greenhouse gas-Air Pollution Interactions and Synergies model was used to provide these estimates. Uncertainties exist in the emission inventories and related input parameters (emission factors, fuel quality), the estimates of emission factors, control potentials, characteristics and uptake of future abatement technologies, enforcement of air quality legislation, the atmospheric dispersion calculations and the impact assessment as well as the models that feed into GAINS. A discussion of uncertainty within the GAINS model is provided in Amann et al. (2011).[23] Modelling of $PM_{2.5}$ concentrations and associated uncertainties are described by Kiesewetter et al.(2015)[24,25] for Europe and for regions outside Europe by Amann et al. (2020).[27]
	Exposure-outcome associations	For regions outside of Europe, disease-specific integrated exposure response (IER) relationships developed within the Global Burden of Disease 2013 study.[29] The disease-specific IER curves and the 95% confidence intervals are presented in Figure S2. However, these ranges are likely to underestimate true uncertainty ranges: In recent years different exposure response relationships have been published which result in much higher attributable mortality numbers (Burnett et al., 2018),[92] while other assessments such as GBD 2017 (GBD Risk Factors collaborators 2018)[93] give estimates similar to ours. The Exposure-response relationships for Europe are applied to all-cause mortality among population over 30 as reported under the REVIHAAP assessment. The risk increase of all-cause mortality per 10$\mu g/m^3$ $PM_{2.5}$ is 6.2% with a 95% confidence interval of 4.1-8.4%.[94]
**Agriculture and diet**	Model uncertainties	Model uncertainties have been described by Springmann et al. (2018)[32,33] and include the values of planetary boundaries, feedback effects between different measures of change and the uptake of technologies that currently have large uncertainties.
	Projections of food demand	Future projections of food demand were income-dependent and followed a middle-of-the-road socio-economic development pathway (shared socio-economic pathway 2, SSP2), as developed by the climate change research community.[36,37] The population and development projections and uncertainties are described in Samir and Lutz (2017).[38] The resulting projections on food demand in this model are in line with other projections of food demand.[39,40]
	Policy uptake of the three scenarios (CPS, SPS, HPS)	These scenarios rely on changes across three areas: technological progress, food loss and waste and dietary change. As presented in Table S1, few NDCs currently specify emissions reductions in the agriculture sector and none specify dietary change, which could yield health co-benefits. There is much uncertainty regarding the policy measures governments will use to address this. A wide range of policy measures are required to improve population dietary habits, including education, labelling, quality standards, economic incentives, availability of food options and school and workplace interventions[95] but all the changes in this model are considered attainable.[32]
	Exposure-outcome associations	The relative risk parameters used in the comparative risk assessment were taken from meta-analyses where the risk-disease associations were graded as moderate or high. The relative risk parameters and 95% confidence intervals are presented in Table S2.
**Transport and physical activity**	Active travel forecasts uncertainties	Data at baseline are likely reasonably robust for the UK and Germany, and to a lesser extent the USA but come with considerable uncertainty for the other countries. National travel survey data was used for each country where available. For Brazil, Indonesia, and Nigeria, no national travel data are available and so data for São Paulo, Greater Jakarta and Lagos were used.

		Percentage of people who are active in India, Indonesia and South Africa was assessed by converting active travel commuting mode share in these countries based on an Indian medium sized city (Visakhapatnam) data. Nigeria's mode share was converted to people who are active based on Accra data.<br><br>There is considerable uncertainty regarding the future travel patterns in all countries. Climate change itself could affect mobility patterns; with increases in temperature in some areas making walking and cycling more difficult and dependent on adaptation (greening) measures. In the CPS/ business as usual case it was assumed that there will be no change in percentage of people who are active in the USA, the UK, Germany and Brazil; whilst, based on available evidence a large decrease in active travel was assumed for China, South Africa, India, Nigeria and Indonesia; but levels could fall further still.<br>The upper limit of the proportion of the population walking 210 minutes or cycling 180 minutes per week was assumed to be 75% for all age groups below 80 and 70% for the population over 80 years, based on active travel patterns in Germany. This is not a strict upper limit and a higher value would have the greatest relative difference in Germany. However, we developed the two future scenarios (HPS, CPS; including the baseline case) which provide a range on health benefits and helps in understanding the impact of uncertainty on results.
	Policy uptake of the three scenarios (CPS, SPS, HPS)	As presented in SI Table 1, few countries mention walking and cycling in their NDCs or related policies and there is uncertainty regarding the policy measures governments will introduce to increase active travel or the effects of such policies on walking and cycling rates.[96]<br>Policies measures include society-level policies (speed limits and fuel prices), regional and city-level policies (including densification and mixed land use), route-level policies (improving accessibility, connectivity, safety and quality of routes) and individual-oriented policies (such as cycle lessons).<br>Countries with high pollution (air and noise) and active travel related injuries (such as India) are likely to result in lower health benefits for the same dose of physical activity as compared to other countries (such as Germany).<br>Policies that simultaneously reduce air pollution[97] and road traffic injury risk will lead to greater benefits and are likely to be more effective at increasing uptake.
	Exposure-outcome associations	Benefits of physical activity are well established with an evidence base that continues to improve with more robust instruments and studies e.g. Pearce et al. (2020).[98] Dose response relationships were taken from the meta-analysis of reduction in all-cause mortality for walking and cycling by Kelly et al. (2014).[87] Risk reduction for an additional 11.25 Metabolically Equivalent Task (MET) hours per week of walking is 0.89 with a 95% confidence interval of 0.83-0.96 and the risk reduction for an additional 11.25 MET hours per week of cycling is 0.90 with a 95% confidence interval of 0.87-0.94. These CIs do not fully represent the underlying uncertainties as the risks are based on largely on self-report that come with considerable measurement error at one point, and exposure is typically measured at only one point in time. Projecting forward the relationship may change over time based on changing disease burdens (including Covid-19), and treatments, including preventive treatment (e.g. a polypill).
**All**	Feedback	These models do not consider feedback that could occur between the emissions that come from different sectors and resulting health outcomes. For example, physical activity is more beneficial without accounting for potential harms from more air pollution. However, the scenarios have been designed with synergies in mind, i.e. air pollution is lower in the SPS alongside increased physical activity. The models, however, are not directly linked. Additionally, the models do not consider climate feedback, such as the effect of climate change on crop yields and freshwater availability, on ambient $PM_{2.5}$ concentrations or the effect of higher temperatures on people's ability to engage in active travel. However, the climate has been included as a forcing driver within both the IEA energy model, the IIASA model and the agricultural model separately.

Section 4: Country evaluations

The following tables provide details and data on each country considered in the paper.

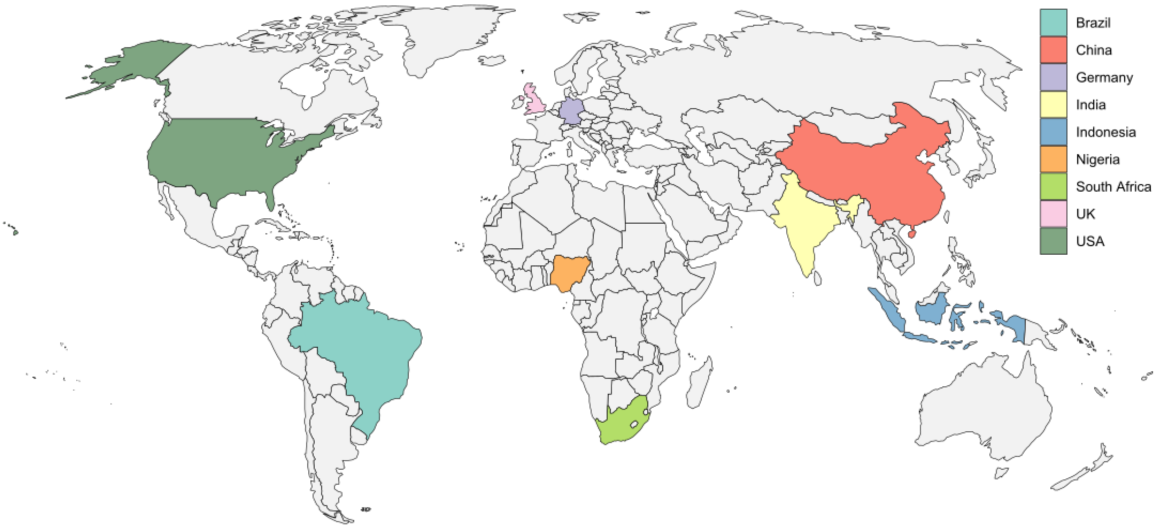

Figure S3. Countries considered in this study

The location of each country considered in this study is given in Figure S3, with relevant characteristics given in Table S5. The countries selected in this study account for 54% of the world population in 2018. The age dependency ratio is defined as the ratio of total working age population (15-64 years) to the total population of all other ages.

Table S5. Country characteristics sourced from the World Bank for the year 2018, unless otherwise stated.[99]

Country Name	Land area (thousand sq. km)	Population (millions)	2040 population projection (millions)	Urban population (% of total population)	Urban population growth (annual %)	Age dependency ratio (% of working-age population)	Life expectancy at birth (years)	GDP per capita, PPP (constant 2017 international $)	Human Development Index
Brazil	8,358	209.5	229.5	86.6	1.1	43.4	75.7	14,596	0.76
China	9,388	1,392.70	1444.3	59.2	2.5	40.4	76.7	15,011	0.74
Germany	349	82.9	76.3	77.3	0.4	54	81	53,660	0.93
India	2,973	1,352.60	1566.7	34	2.3	49.8	69.4	6,538	0.63
Indonesia	1,811	267.7	311.7	55.3	2.3	47.9	71.5	11,370	0.70
Nigeria	910	195.9	350.7	50.3	4.2	87.3	54.3	5,156	0.53
South Africa	1,213	57.8	61.1	66.4	2.1	52.4	63.9	12,631	0.70
United Kingdom	241	66.5	71.0	83.4	0.9	56.4	81.4	46,330	0.92
United States	9,147	326.7	383.4	82.3	0.8	52.7	78.5	61,391	0.92

Table S6. Greenhouse gas emissions and energy use for 2015 baseline year by country

Country Name	Total CO_2 ($GtCO_2$)	Total GHG ($GtCO_2e$)	Total energy use (EJ)	CO_2e/cap (tonne)
Brazil	0.52	1.21	12.9	2.5
China	10.23	12.52	132.4	9.1
Germany	0.70	0.80	12.9	8.6
India	2.17	3.54	31.1	1.7
Indonesia	0.47	0.89	7.4	1.8
Nigeria	0.13	0.39	2.0	0.7
South Africa	0.45	0.54	5.5	8.2
United Kingdom	0.39	0.49	7.6	5.9
United States	5.16	6.55	87.5	16.1

Section 5: Scenario Assumptions and results

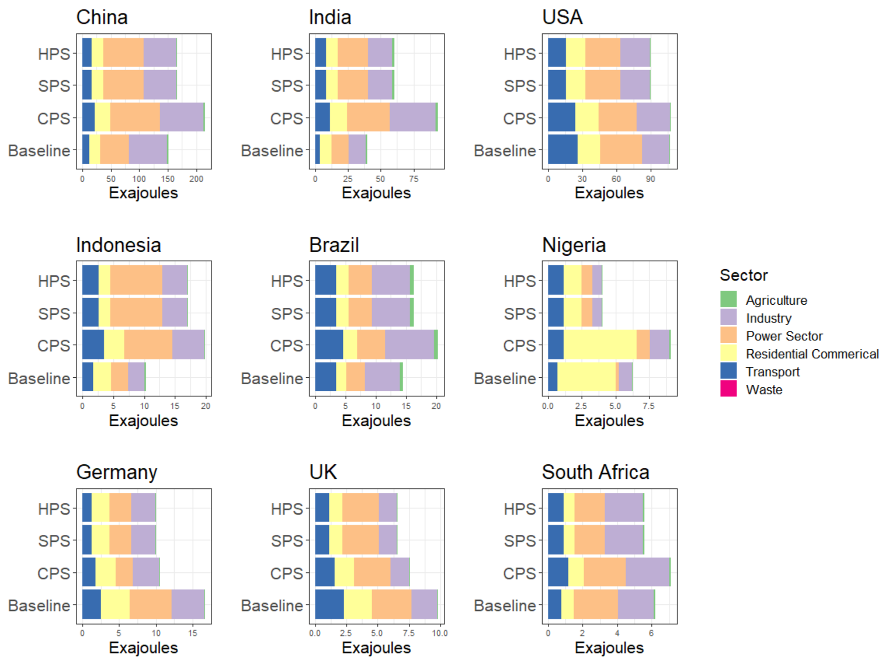

Figure S4. Total primary energy supply (TPES) by sector for each country in 2015 and under future (2040) scenarios

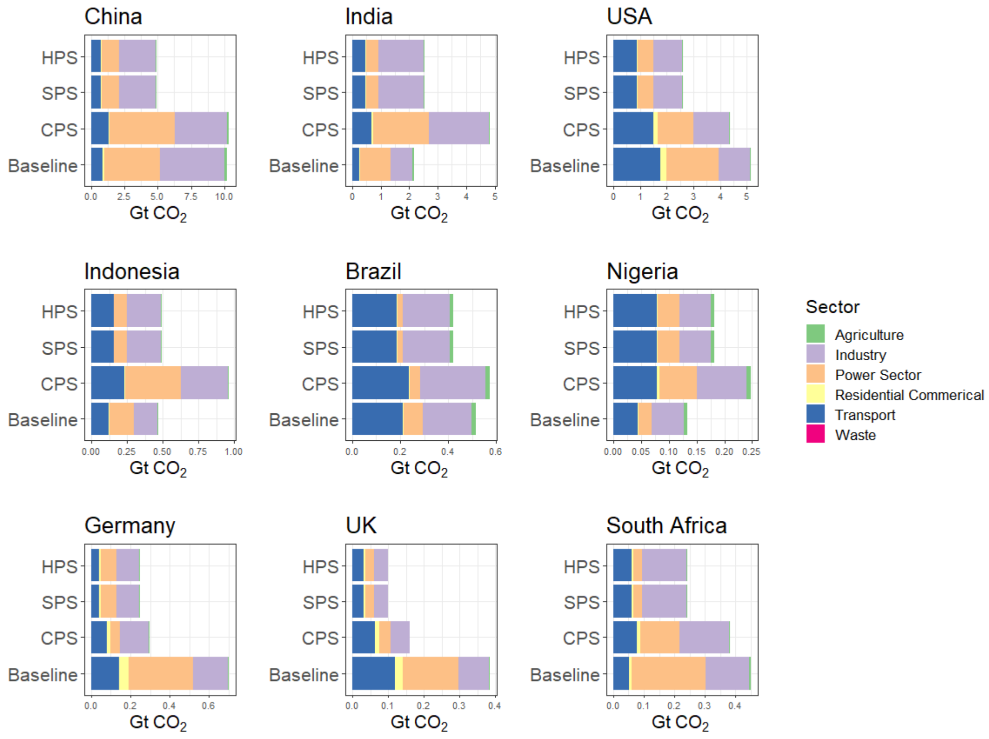

Figure S5. CO_2 emissions by sector for each country in 2015 and under future (2040) scenarios

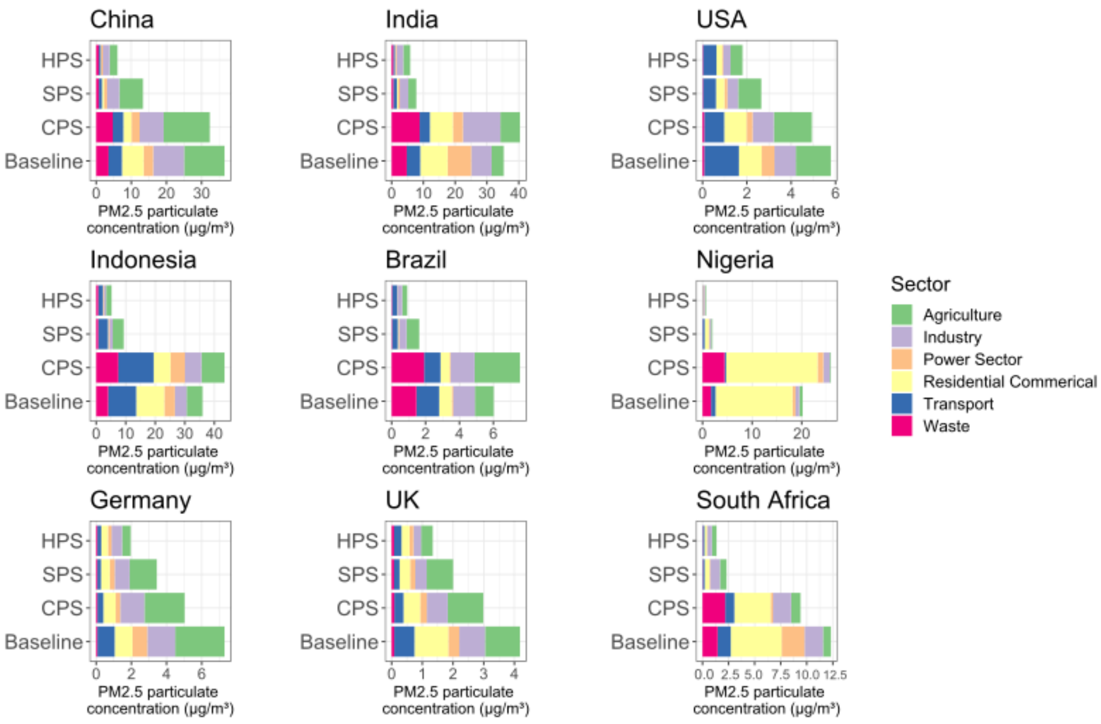

Figure S6. PM2.5 concentration by sector for each country (excluding natural sources)

Table S7: Greenhouse gas emissions, and PM$_{2.5}$ concentration by country and sector.

Country	Sector	Scenario	CO_2 (Gt)	Non-CO_2 GHGs ($GTCO_2e$)	Total ($GHGCO_2e$)	Total Primary energy (EJ)	PM25 concertation (µg/m^3)
**Brazil**	Power Sector	Baseline	0.078	0.002	0.080	3.1	0.09
		CPS	0.045	0.002	0.046	4.6	0.06
		SPS	0.022	0.001	0.023	3.9	0.04
		HPS	0.022	0.001	0.023	3.9	0.02
	Agriculture	Baseline	0.018	0.505	0.523	0.5	1.11
		CPS	0.019	0.583	0.603	0.7	2.68
		SPS	0.017	0.487	0.503	0.6	0.75
		HPS	0.017	0.341	0.358	0.6	0.30
	Transport	Baseline	0.212	0.005	0.217	3.5	1.34
		CPS	0.237	0.007	0.244	4.6	0.97
		SPS	0.186	0.005	0.191	3.5	0.33
		HPS	0.186	0.005	0.191	3.5	0.30
	Residential Commercial	Baseline	0.002	0.004	0.007	1.6	0.73
		CPS	0.003	0.003	0.006	2.4	0.54
		SPS	0.002	0.001	0.002	1.9	0.04
		HPS	0.002	0.001	0.002	1.9	0.01
	Industry	Baseline	0.204	0.021	0.225	5.8	1.29
		CPS	0.271	0.025	0.296	8.1	1.41
		SPS	0.197	0.009	0.206	6.4	0.43
		HPS	0.197	0.009	0.206	6.4	0.27

	Waste	Baseline	0.000	0.080	0.080	0.0	1.46
		CPS	0.001	0.105	0.106	0.0	1.91
		SPS	0.000	0.044	0.044	0.0	0.03
		HPS	0.000	0.044	0.044	0.0	0.03
	Power Sector	Baseline	4.217	0.022	4.239	51.6	2.74
		CPS	4.876	0.033	4.909	87.9	2.33
		SPS	1.296	0.008	1.304	70.2	0.85
		HPS	1.296	0.008	1.304	70.2	0.59
	Agriculture	Baseline	0.192	0.808	1.000	2.3	11.52
		CPS	0.125	0.955	1.080	2.6	13.14
		SPS	0.061	0.652	0.714	1.7	6.73
		HPS	0.061	0.686	0.747	1.7	2.22
	Transport	Baseline	0.824	0.021	0.845	12.2	3.83
		CPS	1.291	0.031	1.322	21.8	2.99
		SPS	0.755	0.019	0.774	16.8	0.92
**China**		HPS	0.755	0.019	0.774	16.8	0.53
	Residential Commercial	Baseline	0.150	0.036	0.186	18.7	6.16
		CPS	0.093	0.035	0.128	27.1	2.33
		SPS	0.033	0.002	0.034	20.7	0.59
		HPS	0.033	0.002	0.034	20.7	0.05
	Industry	Baseline	4.851	0.783	5.635	66.3	8.79
		CPS	3.973	0.796	4.769	76.7	6.83
		SPS	2.746	0.181	2.927	57.1	3.49
		HPS	2.746	0.181	2.927	57.1	1.91
	Waste	Baseline	0.001	0.308	0.309	0.0	3.49
		CPS	0.001	0.573	0.574	0.0	4.73
		SPS	0.000	0.218	0.218	0.0	0.74
		HPS	0.000	0.218	0.218	0.0	0.73
	Power Sector	Baseline	0.328	0.005	0.334	5.7	0.84
		CPS	0.047	0.002	0.049	2.3	0.26
		SPS	0.080	0.001	0.081	2.9	0.28
		HPS	0.080	0.001	0.081	2.9	0.21
	Agriculture	Baseline	0.006	0.062	0.068	0.1	2.80
		CPS	0.003	0.057	0.060	0.1	2.29
		SPS	0.002	0.044	0.046	0.0	1.55
		HPS	0.002	0.040	0.042	0.0	0.49
**Germany**	Transport	Baseline	0.144	0.001	0.145	2.6	0.98
		CPS	0.081	0.001	0.082	1.8	0.34
		SPS	0.042	0.001	0.042	1.3	0.20
		HPS	0.042	0.001	0.042	1.3	0.22
	Residential Commercial	Baseline	0.049	0.004	0.053	3.9	1.01
		CPS	0.019	0.002	0.021	2.8	0.70
		SPS	0.008	0.001	0.009	2.4	0.50
		HPS	0.008	0.001	0.009	2.4	0.38
	Industry	Baseline	0.176	0.010	0.186	4.4	1.59
		CPS	0.147	0.005	0.152	3.7	1.37

		SPS	0.116	0.008	0.123	3.4	0.85
		HPS	0.116	0.008	0.123	3.4	0.59
	Waste	Baseline	0.000	0.009	0.009	0.0	0.07
		CPS	0.000	0.005	0.005	0.0	0.07
		SPS	0.000	0.004	0.004	0.0	0.07
		HPS	0.000	0.004	0.004	0.0	0.07
**India**	Power Sector	Baseline	1.071	0.005	1.076	13.2	7.42
		CPS	1.945	0.011	1.956	32.5	3.18
		SPS	0.444	0.004	0.448	22.8	0.53
		HPS	0.444	0.004	0.448	22.8	0.32
	Agriculture	Baseline	0.055	0.747	0.801	1.0	3.82
		CPS	0.047	0.846	0.894	2.2	6.18
		SPS	0.029	0.641	0.670	1.8	2.51
		HPS	0.029	0.729	0.758	1.8	2.21
	Transport	Baseline	0.246	0.006	0.252	3.6	4.23
		CPS	0.672	0.016	0.688	11.1	3.23
		SPS	0.463	0.010	0.473	8.5	1.16
		HPS	0.463	0.010	0.473	8.5	0.54
**Indonesia**	Residential Commercial	Baseline	0.042	0.055	0.098	8.8	8.61
		CPS	0.065	0.039	0.105	13.3	7.20
		SPS	0.024	0.002	0.026	8.9	0.39
		HPS	0.024	0.002	0.026	8.9	0.20
	Industry	Baseline	0.756	0.095	0.851	13.2	6.43
		CPS	2.107	0.235	2.342	34.7	11.79
		SPS	1.592	0.073	1.665	18.2	2.67
		HPS	1.592	0.073	1.665	18.2	2.08
	Waste	Baseline	0.001	0.228	0.230	0.0	4.77
		CPS	0.001	0.374	0.375	0.0	8.81
		SPS	0.000	0.236	0.236	0.0	0.50
		HPS	0.000	0.236	0.236	0.0	0.50
	Power Sector	Baseline	0.169	0.001	0.171	2.8	3.58
		CPS	0.391	0.003	0.394	7.8	4.88
		SPS	0.089	0.001	0.089	8.5	0.33
		HPS	0.089	0.001	0.089	8.5	0.29
	Agriculture	Baseline	0.004	0.144	0.148	0.2	5.40
		CPS	0.008	0.158	0.166	0.1	7.81
		SPS	0.006	0.115	0.121	0.1	3.69
		HPS	0.006	0.119	0.126	0.1	1.87
	Transport	Baseline	0.123	0.004	0.127	1.7	9.49
		CPS	0.234	0.005	0.239	3.5	11.99
		SPS	0.157	0.004	0.161	2.7	3.32
		HPS	0.157	0.004	0.161	2.7	1.56
	Residential Commercial	Baseline	0.003	0.017	0.020	2.9	9.52
		CPS	0.002	0.009	0.010	3.2	5.71
		SPS	0.001	0.000	0.002	1.8	0.12
		HPS	0.001	0.000	0.002	1.8	0.01

	Industry	Baseline	0.167	0.147	0.314	2.6	4.00
		CPS	0.328	0.174	0.501	5.2	5.60
		SPS	0.240	0.028	0.269	4.0	1.20
		HPS	0.240	0.028	0.269	4.0	0.82
	Waste	Baseline	0.001	0.055	0.056	0.0	4.03
		CPS	0.000	0.101	0.102	0.0	7.46
		SPS	0.000	0.046	0.046	0.0	0.66
		HPS	0.000	0.046	0.046	0.0	0.66
	Power Sector	Baseline	0.024	0.001	0.025	0.3	0.62
		CPS	0.068	0.001	0.069	1.0	1.17
		SPS	0.039	0.000	0.039	0.8	0.19
		HPS	0.039	0.000	0.039	0.8	0.07
	Agriculture	Baseline	0.006	0.079	0.085	0.0	0.66
		CPS	0.008	0.086	0.093	0.1	0.21
		SPS	0.006	0.071	0.077	0.1	0.26
		HPS	0.006	0.075	0.081	0.1	0.18
	Transport	Baseline	0.044	0.001	0.045	0.7	0.92
		CPS	0.078	0.002	0.081	1.1	0.32
		SPS	0.078	0.001	0.079	1.2	0.40
**Nigeria**		HPS	0.078	0.001	0.079	1.2	0.08
	Residential Commercial	Baseline	0.001	0.041	0.042	4.3	15.50
		CPS	0.005	0.046	0.050	5.4	18.43
		SPS	0.002	0.003	0.004	1.3	0.80
		HPS	0.002	0.003	0.004	1.3	0.12
	Industry	Baseline	0.057	0.101	0.158	1.0	0.78
		CPS	0.089	0.109	0.198	1.4	1.31
		SPS	0.057	0.009	0.066	0.7	0.33
		HPS	0.057	0.009	0.066	0.7	0.26
	Waste	Baseline	0.000	0.018	0.018	0.0	1.71
		CPS	0.000	0.037	0.037	0.0	4.44
		SPS	0.000	0.023	0.023	0.0	0.03
		HPS	0.000	0.023	0.023	0.0	0.03
	Power Sector	Baseline	0.245	0.001	0.246	2.6	2.19
		CPS	0.129	0.001	0.130	2.4	0.23
		SPS	0.027	0.000	0.028	1.7	0.05
		HPS	0.027	0.000	0.028	1.7	0.03
	Agriculture	Baseline	0.006	0.029	0.035	0.1	0.75
		CPS	0.003	0.036	0.039	0.1	0.90
**South Africa**		SPS	0.002	0.031	0.033	0.1	0.64
		HPS	0.002	0.031	0.033	0.1	0.45
	Transport	Baseline	0.051	0.001	0.053	0.8	1.26
		CPS	0.076	0.001	0.078	1.2	0.88
		SPS	0.059	0.001	0.060	0.9	0.18
		HPS	0.059	0.001	0.060	0.9	0.16
	Residential Commercial	Baseline	0.007	0.004	0.012	0.7	4.89
		CPS	0.012	0.002	0.014	0.9	3.49

		SPS	0.008	0.000	0.008	0.6	0.47
		HPS	0.008	0.000	0.008	0.6	0.23
	Industry	Baseline	0.142	0.024	0.166	2.1	1.78
		CPS	0.163	0.019	0.182	2.6	1.74
		SPS	0.146	0.010	0.156	2.2	0.95
		HPS	0.146	0.010	0.156	2.2	0.46
	Waste	Baseline	0.000	0.014	0.014	0.0	1.45
		CPS	0.000	0.020	0.020	0.0	2.17
		SPS	0.000	0.009	0.009	0.0	0.02
		HPS	0.000	0.009	0.009	0.0	0.02
UK	Power Sector	Baseline	0.157	0.002	0.160	3.2	0.36
		CPS	0.032	0.002	0.033	2.9	0.22
		SPS	0.025	0.001	0.026	2.9	0.17
		HPS	0.025	0.001	0.026	2.9	0.13
	Agriculture	Baseline	0.003	0.052	0.055	0.1	1.14
		CPS	0.001	0.049	0.051	0.0	1.16
		SPS	0.001	0.041	0.042	0.0	0.86
		HPS	0.001	0.039	0.040	0.0	0.38
	Transport	Baseline	0.118	0.001	0.119	2.3	0.66
		CPS	0.064	0.001	0.065	1.6	0.30
		SPS	0.032	0.000	0.033	1.1	0.18
		HPS	0.032	0.000	0.033	1.1	0.24
	Residential Commercial	Baseline	0.022	0.004	0.026	2.3	1.10
		CPS	0.011	0.001	0.012	1.6	0.54
		SPS	0.004	0.000	0.004	1.1	0.34
		HPS	0.004	0.000	0.004	1.1	0.26
	Industry	Baseline	0.084	0.009	0.093	2.0	0.84
		CPS	0.053	0.003	0.056	1.5	0.68
		SPS	0.038	0.004	0.043	1.4	0.37
		HPS	0.038	0.004	0.043	1.4	0.25
	Waste	Baseline	0.001	0.018	0.018	0.0	0.08
		CPS	0.000	0.014	0.014	0.0	0.09
		SPS	0.000	0.007	0.007	0.0	0.08
		HPS	0.000	0.007	0.007	0.0	0.08
USA	Power Sector	Baseline	1.959	0.015	1.974	36.9	0.57
		CPS	1.336	0.014	1.350	33.0	0.27
		SPS	0.579	0.003	0.581	31.0	0.13
		HPS	0.579	0.003	0.581	31.0	0.04
	Agriculture	Baseline	0.052	0.428	0.480	0.9	1.58
		CPS	0.037	0.452	0.489	0.7	1.72
		SPS	0.020	0.332	0.353	0.7	1.04
		HPS	0.020	0.275	0.295	0.7	0.54
	Transport	Baseline	1.772	0.041	1.813	25.7	1.57
		CPS	1.492	0.037	1.529	23.6	0.90
		SPS	0.871	0.023	0.894	16.1	0.59
		HPS	0.871	0.023	0.894	16.1	0.61

Residential Commercial	Baseline	0.211	0.029	0.240	20.0	1.03
	CPS	0.167	0.027	0.194	20.8	1.01
	SPS	0.058	0.002	0.060	16.6	0.38
	HPS	0.058	0.002	0.060	16.6	0.26
Industry	Baseline	1.169	0.511	1.680	24.0	0.96
	CPS	1.336	0.603	1.939	29.8	0.96
	SPS	1.093	0.330	1.423	25.7	0.49
	HPS	1.093	0.330	1.423	25.7	0.34
Waste	Baseline	0.001	0.180	0.182	0.0	0.07
	CPS	0.001	0.215	0.215	0.0	0.07
	SPS	0.000	0.083	0.083	0.0	0.02
	HPS	0.000	0.083	0.083	0.0	0.02

**Table S8. Avoided deaths due exposure to ambient $PM_{2.5}$, by sector**		Deaths Avoided		Deaths Avoided Per 100,000 population	
		SPS	HPS	SPS	HPS
Brazil	Agriculture	4001	5888	2	3
Brazil	Industry	4746	5601	2	2
Brazil	Power Sector	137	227	0	0
Brazil	Residential-Commercial	1619	1665	1	1
Brazil	Transport	2521	2996	1	1
Brazil	Waste	8045	8079	3	3
China	Agriculture	157434	379735	11	27
China	Industry	68026	151068	5	11
China	Power Sector	39469	51419	3	4
China	Residential-Commercial	48898	68956	3	5
China	Transport	56528	71456	4	5
China	Waste	133112	133173	9	9
Germany	Agriculture	4743	9570	6	12
Germany	Industry	2670	3941	3	5
Germany	Power Sector	-110	217	0	0
Germany	Residential-Commercial	921	1447	1	2
Germany	Transport	537	430	1	1
Germany	Waste	9	9	0	0
India	Agriculture	26435	48508	2	3
India	Industry	121106	141434	8	9
India	Power Sector	36441	41711	2	3
India	Residential-Commercial	103062	104951	6	7
India	Transport	15670	25547	1	2
India	Waste	130835	129605	8	8
Indonesia	Agriculture	15741	26263	5	8
Indonesia	Industry	21523	25123	7	8
Indonesia	Power Sector	16905	17668	5	6
Indonesia	Residential-Commercial	25352	25808	8	8
Indonesia	Transport	25224	37118	8	12
Indonesia	Waste	25796	27149	8	9
Nigeria	Agriculture	1181	1362	0	0
Nigeria	Industry	1618	1813	0	1
Nigeria	Power Sector	1650	1952	0	1
Nigeria	Residential-Commercial	31780	33345	10	10
Nigeria	Transport	-480	355	0	0
Nigeria	Waste	8090	8088	2	2
South Africa	Agriculture	1105	1390	2	2

South Africa	Industry	1296	1752	2	3
South Africa	Power Sector	193	211	0	0
South Africa	Residential-Commercial	3112	3328	5	5
South Africa	Transport	584	649	1	1
South Africa	Waste	2119	2127	3	3
United Kingdom	Agriculture	1188	2939	2	4
United Kingdom	Industry	1145	1572	2	2
United Kingdom	Power Sector	154	282	0	0
United Kingdom	Residential-Commercial	664	905	1	1
United Kingdom	Transport	283	49	0	0
United Kingdom	Waste	24	24	0	0
United States	Agriculture	11429	14315	3	4
United States	Industry	6398	7496	2	2
United States	Power Sector	1654	2045	0	1
United States	Residential-Commercial	6332	7014	2	2
United States	Transport	4228	4953	1	1
United States	Waste	519	548	0	0

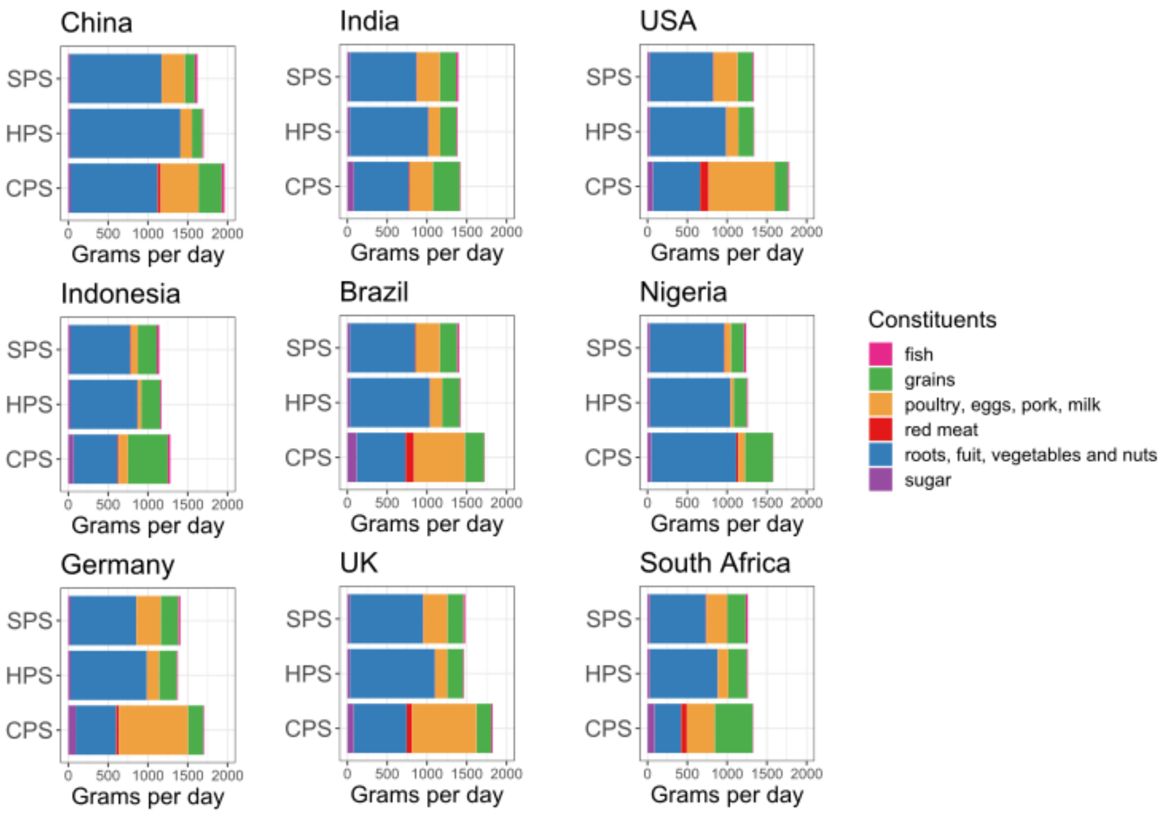

Figure S7. Composition of diets in terms of grams per day by country and scenario

Table S9. Dietary composition in grams per day by scenario and country.

Country	Scenario	Total	grains	roots, fruit, vegetables and nuts	sugar	red meat	poultry, eggs, pork, milk	fish
	CPS	1,726	245	615	117	107	636	6
	SPS	1,405	220	824	31	10	296	24
Brazil	HPS	1,422	220	1,006	31	5	148	12
	CPS	1,961	294	1,090	32	41	473	32
	SPS	1,623	130	1,140	30	4	286	32
China	HPS	1,700	130	1,378	30	2	143	16
	CPS	1,704	192	509	93	34	866	11
	SPS	1,407	225	821	31	3	303	24
Germany	HPS	1,376	225	956	31	1	152	12
	CPS	1,422	340	694	81	11	286	9
	SPS	1,395	208	835	31	11	284	26
India	HPS	1,387	208	987	31	5	142	13
	CPS	1,280	502	553	67	15	113	31
	SPS	1,142	239	749	31	10	84	31
Indonesia	HPS	1,170	239	838	31	5	42	15
	CPS	1,580	352	1,069	47	26	82	4
	SPS	1,239	167	931	31	10	74	26
Nigeria	HPS	1,262	167	1,009	31	5	37	13
	CPS	1,327	478	335	88	71	349	6
	SPS	1,261	235	702	31	10	257	26
South Africa	HPS	1,262	235	850	31	5	128	13
	CPS	1,827	191	665	80	68	807	17
	SPS	1,480	204	919	31	7	299	21
UK	HPS	1,467	204	1,069	31	4	149	10
	CPS	1,781	169	593	68	108	829	14
	SPS	1,338	194	793	31	9	297	14
USA	HPS	1,341	194	956	31	4	149	7

Table S10. Deaths avoided by risk factor, scenario and country. Note: The health impacts associated with the combination of all risks is smaller than the sum of individual risks, because the former controls for co-exposure, i.e. each death is attributed to one risk factor only.

Country	Diet scenario	Weight risk factors	Dietary risk factors	Underweight	Overweight	Obese	Low fruit	Low vegetables	Low nuts seeds	Low legumes	Low fish	High red meat	All risk factors
Brazil	SPS	211,760	158,210	7,440	46,060	158,260		59,160	3,520	9,020	17,780	82,950	328,040
Brazil	HPS	211,760	170,080	7,440	46,060	158,260	9,780	57,370	3,520	23,050	8,890	87,070	336,270
China	SPS	899,940	1,663,060	293,450	317,060	289,440	444,770		107,220	270,680		963,780	2,409,640
China	HPS	899,940	2,106,030	293,450	317,060	289,440	485,540	344,510	107,220	433,920		1,008,910	2,810,400
Germany	SPS	75,930	88,430	1,140	18,620	56,170	8,320	22,760	11,810	19,710	7,250	34,040	143,770
Germany	HPS	75,930	89,050	1,140	18,620	56,170	9,180	19,940	11,810	25,720	3,620	35,540	143,710
India	SPS	1,073,890	764,200	630,850	175,960	267,080	247,410	24,560	225,590	183,580	148,510		1,741,860
India	HPS	1,073,890	910,390	630,850	175,960	267,080	278,130	113,160	225,590	287,890	74,260	30,730	1,869,300
Indonesia	SPS	209,780	107,410	44,040	62,120	103,630	5,710	58,220		40,690		9,030	301,970
Indonesia	HPS	209,780	130,500	44,040	62,120	103,630	17,460	55,720		48,710		17,820	321,630
Nigeria	SPS	59,950	34,270	9,380	15,720	34,850	1,610	7,940	5,000	7,090	8,300	7,550	88,490
Nigeria	HPS	59,950	37,860	9,380	15,720	34,850	4,410	8,370	5,000	9,450	4,150	9,800	91,550
South Africa	SPS	65,660	45,800	2,350	7,440	55,870	12,720	10,430	7,470	5,740	3,320	13,960	97,160
South Africa	HPS	65,660	48,400	2,350	7,440	55,870	14,360	10,790	7,470	7,820	1,660	15,060	98,900
UK	SPS	62,510	48,300	1,320	11,490	49,700		15,880	5,880	12,200	1,780	18,810	98,420
UK	HPS	62,510	51,090	1,320	11,490	49,700	2,820	13,610	5,880	16,130	890	19,870	100,100
USA	SPS	473,340	276,240	6,720	55,840	410,780	17,030	45,320	13,310	75,710	3,110	150,700	654,580
USA	HPS	473,340	294,310	6,720	55,840	410,780	25,140	35,060	13,310	101,030	1,560	156,530	664,050

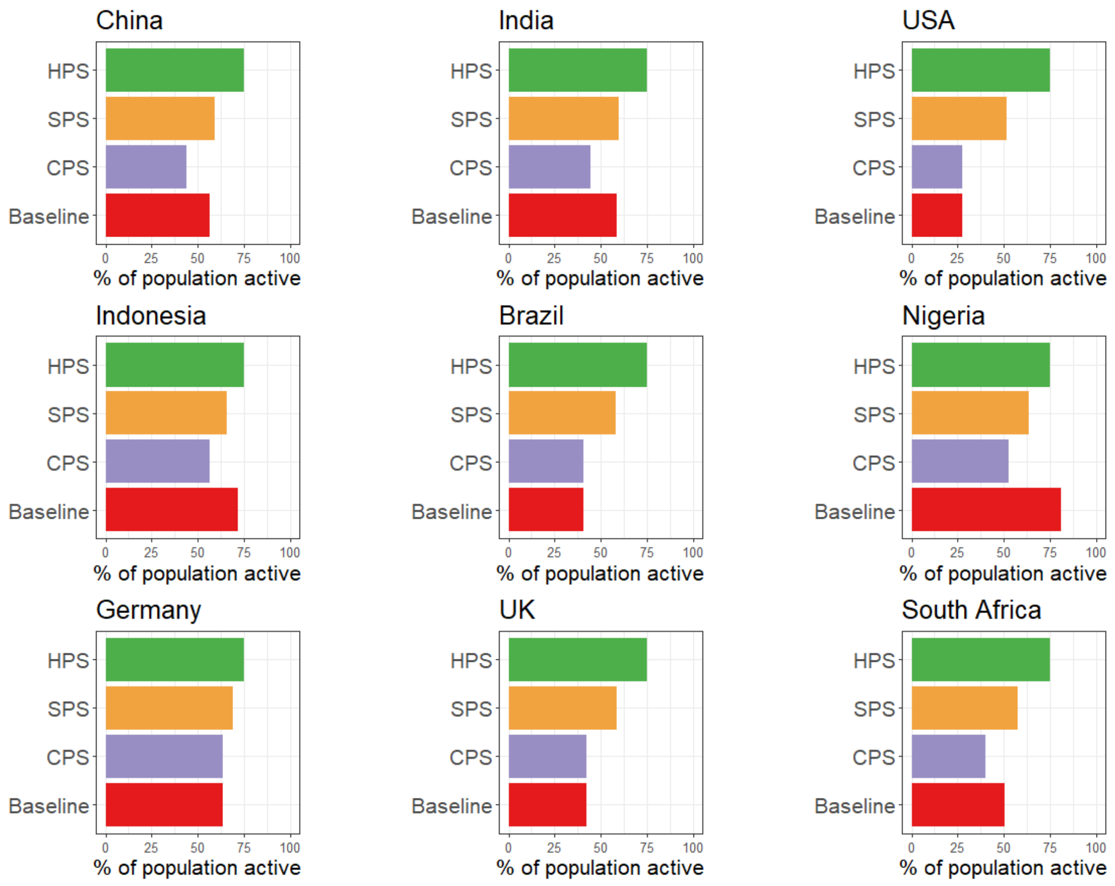

Figure S8. Percentage of the population either walking or cycling on a weekly basis, by country and scenario.

Table S11. Estimates for active travel participation by country and scenario.

	Scenario	Brazil	China	Germany	Indonesia	India	Nigeria	South Africa	UK	USA
% of people who walk or cycle	Baseline	0.41	0.56	0.64	0.72	0.59	0.81	0.51	0.42	0.28
% of people who walk		0.39	0.39	0.43	0.71	0.52	0.80	0.50	0.39	0.26
% of people who cycle		0.03	0.28	0.36	0.03	0.14	0.06	0.02	0.05	0.03
Active Travel in 2040 - % of people who walk or cycle	CPS	0.41	0.44	0.64	0.56	0.45	0.53	0.40	0.42	0.28
	SPS	0.58	0.59	0.69	0.66	0.60	0.64	0.58	0.59	0.51
	HPS	0.75	0.75	0.75	0.75	0.75	0.75	0.75	0.75	0.75
Walking in 2040 - % people doing walking (as main-mode)	CPS	0.39	0.39	0.43	0.56	0.41	0.50	0.39	0.39	0.26
	SPS	0.45	0.44	0.45	0.59	0.46	0.53	0.45	0.45	0.34
	HPS	0.50	0.50	0.47	0.62	0.51	0.57	0.51	0.50	0.41
Walking in 2040 - Net additional people: walking	SPS (km; in millions)	13	75	2	10	79	13	4	3	28
	HPS (km; in millions)	26	149	3	19	158	26	7	7	56
Walking in 2040 - Net walking km	SPS (km; in millions)	8,054	46,595	940	5,994	49,323	8,198	2,192	2,126	17,518
	HPS (km; in millions)	16,107	93,189	1,880	11,988	98,646	16,395	4,384	4,253	35,036
Cycling in 2040 - % people doing walking (as main-mode)	CPS	0.03	0.08	0.36	0.01	0.06	0.06	0.02	0.05	0.03
	SPS	0.14	0.18	0.40	0.07	0.16	0.13	0.13	0.16	0.19
	HPS	0.26	0.28	0.44	0.13	0.26	0.21	0.25	0.27	0.34
Cycling in 2040 - Net additional people: cycling	SPS (km; in millions)	26	148	3	19	158	26	7	7	58
	HPS (km; in millions)	51	297	6	38	315	53	14	15	116
Cycling in 2040 - Net cycling km	SPS (km; in millions)	39,987	231,372	4,474	29,853	245,930	40,967	10,931	11,577	90,371
	HPS (km; in millions)	79,974	462,743	8,947	59,707	491,859	81,935	21,861	23,155	180,742

Table S12. Reduction in relative risk for each scenario for each country for given age group for active travel

		Reduction in Relative Risk (relative to Baseline 2018)		
Country	Age	CPS	SPS	HPS
China	<60	-0.03	0.04	0.07
	60-70	-0.03	0.04	0.07
	70-80	-0.03	0.04	0.07
	80+	-0.03	0.03	0.06
Brazil	<60	0.00	0.04	0.07
	60-70	0.00	0.04	0.07
	70-80	0.00	0.04	0.07
	80+	0.00	0.04	0.07
Germany	<60	0.00	0.01	0.03
	60-70	0.00	0.01	0.01
	70-80	0.00	0.00	0.01
	80+	0.00	0.01	0.01
India	<60	-0.03	0.04	0.06
	60-70	-0.03	0.03	0.06
	70-80	-0.03	0.03	0.06
	80+	-0.03	0.03	0.06
Indonesia	<60	-0.03	0.02	0.04
	60-70	-0.03	0.02	0.04
	70-80	-0.03	0.02	0.04
	80+	-0.03	0.02	0.03
Nigeria	<60	-0.05	0.02	0.05
	60-70	-0.05	0.02	0.05
	70-80	-0.05	0.02	0.05
	80+	-0.05	0.02	0.04
South Africa	<60	-0.02	0.04	0.08
	60-70	-0.02	0.04	0.08
	70-80	-0.02	0.04	0.08
	80+	-0.02	0.04	0.07
UK	<60	0.00	0.03	0.06
	60-70	0.00	0.04	0.07
	70-80	0.00	0.05	0.09
	80+	0.00	0.07	0.12
USA	<60	0.00	0.06	0.11
	60-70	0.00	0.06	0.10
	70-80	0.00	0.07	0.11
	80+	0.00	0.08	0.14

Table S13. Deaths avoided by active travel scenario

	Deaths Avoided by scenario		Deaths Avoided per 100,000 population	
Country	SPS	HPS	SPS	HPS
Brazil	56,224	102,386	24	45
China	440,757	809,324	31	56
Germany	2,856	5,631	4	7
India	364,948	670,230	23	43
Indonesia	37,759	71,762	12	23
Nigeria	29,376	55,094	8	16
South Africa	19,341	35,011	32	57
UK	21,486	38,441	30	54
USA	172,618	300,419	45	78
Total	1,145,365	2,088,298	209	379

References

1. Climate Action Tracker. Current Policy Tracker. 2020. https://climateactiontracker.org/countries/indonesia/current-policy-projections/.
2. UNFCCC. NDC registry. 2020. https://www4.unfccc.int/sites/NDCStaging/Pages/All.aspx.
3. IEA. World Energy Model Documentation. Paris, France: International Energy Agency, 2019.
4. Rafaj P, Kiesewetter G, Gül T, et al. Outlook for clean air in the context of sustainable development goals. *Global Environmental Change* 2018; **53**: 1-11.
5. Rafaj P, Schöpp W, Russ P, Heyes C, Amann M. Co-benefits of post-2012 global climate mitigation policies. *Mitig Adapt Strateg Glob Change* 2013; **18**(6): 801-24.
6. Amann M, Purohit P, Bhanarkar AD, et al. Managing future air quality in megacities: A case study for Delhi. *Atmospheric Environment* 2017; **161**: 99-111.
7. Bhanarkar AD, Purohit P, Rafaj P, et al. Managing future air quality in megacities: Co-benefit assessment for Delhi. *Atmospheric Environment* 2018; **186**: 158-77.
8. Cofala J, Bertok I, Borken-Kleefeld J, et al. Implications of energy trajectories from the World Energy Outlook 2015 for India's air pollution. Final Report submitted to the International Energy Agency. Paris, France, 2015.
9. Purohit P, Amann M, Mathur R, et al. GAINS-Asia. Scenarios for cost-effective control of air pollution and greenhouse gases in India. Laxenburg, Austria, 2010.
10. Klimont Z, Kupiainen K, Heyes C, et al. Global anthropogenic emissions of particulate matter including black carbon. *Atmospheric Chemistry and Physics* 2017; **17**(14): 8681–723.
11. Global Energy Assessment Writing Team. Global Energy Assessment - Toward a Sustainable Future. Cambridge University Press, Cambridge, UK and New York, NY, USA and the International Institute for Applied Systems Analysis, Laxenburg, Austria; 2012.
12. Amann M, Bertok I, Borken J, et al. GAINS: Potentials and costs for greenhouse gas mitigation in annex i countries. Laxenburg, Austria: International Institute for Applied Systems Analysis 2008.
13. Höglund-Isaksson L, Gómez-Sanabria A, Klimont Z, Rafaj P, Schöpp W. Technical potentials and costs for reducing global anthropogenic methane emissions in the 2050 timeframe–results from the GAINS model. *Environmental Research Communications* 2020; **2**(2): 025004.
14. Purohit P, Höglund Isaksson L. Global emissions of fluorinated greenhouse gases 2005-2050 with abatement potentials and costs. *Atmospheric Chemistry and Physics* 2017; **17**: 2795-816.
15. Winiwarter W, Höglund-Isaksson L, Klimont Z, Schöpp W, Amann M. Technical opportunities to reduce global anthropogenic emissions of nitrous oxide. *Environmental Research Letters* 2018; **13**(1): 014011.
16. IEA. World Energy Outlook 2018. Paris, France: International Energy Agency, 2018.
17. Alexandratos N, Bruisma J. World Agricultural Trends Towards 2030/2050: the 2012 Revision. Rome, Italy: Food and Agriculture Organization of the United Nations, 2012.
18. The Food and Land Use Coalition. Growing Better: Ten Critical Transitions to Transform Food and Land Use. London, UK: The Food and Land Use Coalition, 2019.
19. Höglund-Isaksson L, Purohit P, Amann M, et al. Cost estimates of the Kigali Amendment to phase-down hydrofluorocarbons. *Environmental Science & Policy* 2017; **75**: 138-47.
20. Fang X, Velders GJ, Ravishankara A, Molina MJ, Hu J, Prinn RG. Hydrofluorocarbon (HFC) emissions in China: an inventory for 2005–2013 and projections to 2050. *Environmental science & technology* 2016; **50**(4): 2027-34.
21. Climate & Clean Air Coalition. HFC inventories. 2020. https://www.ccacoalition.org/en/activity/hfc-inventories.
22. IEA. World Energy Outlook 2017. Paris, France: International Energy Agency; 2017.
23. Amann M, Bertok I, Borken-Kleefeld J, et al. Cost-effective control of air quality and greenhouse gases in Europe: Modeling and policy applications. *Environmental Modelling & Software* 2011; **26**(12): 1489-501.
24. Kiesewetter G, Borken-Kleefeld J, Schopp W, et al. Modelling street level PM10 concentrations across Europe: source apportionment and possible futures. 2015.

25. Kiesewetter G, Schoepp W, Heyes C, Amann M. Modelling PM2.5 impact indicators in Europe: Health effects and legal compliance. *Environmental Modelling & Software* 2015; **74**: 201-11.
26. Menut L, Bessagnet B, Khvorostyanov D, et al. CHIMERE 2013: a model for regional atmospheric composition modelling. *Geoscientific Model Development* 2013; **6**: 981–1028.
27. Amann M, Kiesewetter G, Schöpp W, et al. Reducing global air pollution: the scope for further policy interventions. *Philosophical Transactions of the Royal Society A* 2020; **20190331**.
28. WHO. Ambient Air Pollution: A global assessment of exposure and burden of disease. Geneva, Switzerland: World Health Organization, 2016.
29. Forouzanfar MH, Alexander L, Anderson HR, et al. Global, regional, and national comparative risk assessment of 79 behavioural, environmental and occupational, and metabolic risks or clusters of risks in 188 countries, 1990–2013: a systematic analysis for the Global Burden of Disease Study 2013. *The Lancet* 2015; **386**(10010): 2287-323.
30. UNDESA. World Population Prospects: The 2017 revision. New York, NY, USA: United Nations Department of Economic and Social Affairs; 2017.
31. WHO European Centre for Environment and Health. Review of evidence on health aspects of air pollution - REVIHAAP Project. Copenhagen, Denmark: WHO Regional Office for Europe, 2013.
32. Springmann M, Clark M, Mason-D'Croz D, et al. Options for keeping the food system within environmental limits. *Nature* 2018; **562**(7728): 519-25.
33. Springmann M, Wiebe K, Mason-D'Croz D, Sulser TB, Rayner M, Scarborough P. Health and nutritional aspects of sustainable diet strategies and their relationship to environmental impacts – a comparative global modelling analysis with country-level detail. *The Lancet Planetary Health* 2018; **2**: e451-e61.
34. Gustavsson J, Cederberg C, Sonesson U, Van Otterdijk R, Meybeck A. Global food losses and food waste: extent, causes and prevention. Rome: FAO Rome, 2011.
35. Robinson S, Mason-D'Croz D, Islam S, et al. The International Model for Policy Analysis of Agricultural Commodities and Trade (IMPACT) -- Model description for version 3. International Food Policy Research Institute; 2015.
36. Chateau J, Dellink R, Lanzi E, Magné B. Long-term economic growth and environmental pressure: reference scenarios for future global projections. 2012.
37. O'Neill BC, Kriegler E, Riahi K, et al. A new scenario framework for climate change research: the concept of shared socioeconomic pathways. *Climatic change* 2014; **122**(3): 387-400.
38. Samir K, Lutz W. The human core of the shared socioeconomic pathways: Population scenarios by age, sex and level of education for all countries to 2100. *Global Environmental Change* 2017; **42**: 181-92.
39. Alexandratos N, Bruinsma J. World agriculture towards 2030/2050: the 2012 revision. 2012.
40. Valin H, Sands RD, Van der Mensbrugghe D, et al. The future of food demand: understanding differences in global economic models. *Agricultural Economics* 2014; **45**(1): 51-67.
41. Springmann M, Mason-D'Croz D, Robinson S, et al. Global and regional health effects of future food production under climate change: a modelling study. *The Lancet* 2016; **387**(10031): 1937-46.
42. Murray CJL, Ezzati M, Lopez AD, Rodgers A, Vander Hoorn S. Comparative quantification of health risks: conceptual framework and methodological issues. *Population Health Metrics* 2003; **1**(1): 1.
43. Lim SS, Vos T, Flaxman AD, et al. A comparative risk assessment of burden of disease and injury attributable to 67 risk factors and risk factor clusters in 21 regions, 1990–2010: a systematic analysis for the Global Burden of Disease Study 2010. *The Lancet* 2012; **380**(9859): 2224-60.
44. Murray CJL, Ezzati M, Flaxman AD, et al. GBD 2010: design, definitions, and metrics. *Lancet* 2012; **380**(9859): 2063-6.
45. Lozano R, Naghavi M, Foreman K, et al. Global and regional mortality from 235 causes of death for 20 age groups in 1990 and 2010: a systematic analysis for the Global Burden of Disease Study 2010. *The Lancet* 2012; **380**(9859): 2095-128.

46. UNDESA. World Population Prospects: The 2012 Revision. New York, NY, USA: Population Division of the Department of Economic and Social Affairs of the United Nations Secretariat

2013.
47. NCD Risk Factor Collaboration. Trends in adult body-mass index in 200 countries from 1975 to 2014: a pooled analysis of 1698 population-based measurement studies with 19·2 million participants. *The Lancet* 2016; **387**(10026): 1377-96.
48. Afshin A, Micha R, Khatibzadeh S, Mozaffarian D. Consumption of nuts and legumes and risk of incident ischemic heart disease, stroke, and diabetes: a systematic review and meta-analysis. *The American Journal of Clinical Nutrition* 2014; **100**(1): 278-88.
49. Aune D, Giovannucci E, Boffetta P, et al. Fruit and vegetable intake and the risk of cardiovascular disease, total cancer and all-cause mortality—a systematic review and dose-response meta-analysis of prospective studies. *International Journal of Epidemiology* 2017; **46**(3): 1029-56.
50. Aune D, Keum N, Giovannucci E, et al. Nut consumption and risk of cardiovascular disease, total cancer, all-cause and cause-specific mortality: a systematic review and dose-response meta-analysis of prospective studies. *BMC medicine* 2016; **14**(1): 207.
51. Bechthold A, Boeing H, Schwedhelm C, et al. Food groups and risk of coronary heart disease, stroke and heart failure: A systematic review and dose-response meta-analysis of prospective studies. *Critical Reviews in Food Science and Nutrition* 2019; **59**(7): 1071-90.
52. Global BMI Mortality Collaboration, Di Angelantonio E, Bhupathiraju S, et al. Body-mass index and all-cause mortality: individual-participant-data meta-analysis of 239 prospective studies in four continents. *The Lancet* 2016; **388**(10046): 776-86.
53. Schwingshackl L, Hoffmann G, Lampousi AM, et al. Food groups and risk of type 2 diabetes mellitus: a systematic review and meta-analysis of prospective studies. *European Journal of Epidemiology* 2017; **32**(5): 363-75.
54. Zheng J, Huang T, Yu Y, Hu X, Yang B, Li D. Fish consumption and CHD mortality: an updated meta-analysis of seventeen cohort studies. *Public Health Nutrition* 2012; **15**(4): 725-37.
55. Singh GM, Danaei G, Farzadfar F, et al. The Age-Specific Quantitative Effects of Metabolic Risk Factors on Cardiovascular Diseases and Diabetes: A Pooled Analysis. *PLOS ONE* 2013; **8**(7): e65174-e.
56. Micha R, Shulkin ML, Peñalvo JL, et al. Etiologic effects and optimal intakes of foods and nutrients for risk of cardiovascular diseases and diabetes: Systematic reviews and meta-analyses from the Nutrition and Chronic Diseases Expert Group (NutriCoDE). *PLOS ONE* 2017; **12**(4): e0175149-e.
57. GBD Diet Collaborators, Afshin A, Sur PJ, et al. Health effects of dietary risks in 195 countries, 1990-2017: a systematic analysis for the Global Burden of Disease Study 2017. *The Lancet* 2019; **393**(10184): 1958-72.
58. Schwingshackl L, Knüppel S, Schwedhelm C, et al. Perspective: NutriGrade: A Scoring System to Assess and Judge the Meta-Evidence of Randomized Controlled Trials and Cohort Studies in Nutrition Research. *Advances in Nutrition: An International Review Journal* 2016; **7**(6): 994-1004.
59. Schwingshackl L, Schwedhelm C, Hoffmann G, et al. Food groups and risk of colorectal cancer. *International Journal of Cancer* 2018; **142**(9): 1748-58.
60. World Cancer Research Fund/American Institute for Cancer Research. Diet, Nutrition, Physical Activity and Cancer: A Global Perspective. Continuous Update Project Expert Report. London, UK: World Cancer Research Fund International, 2018.
61. Satija A, Yu E, Willett WC, Hu FB. Understanding Nutritional Epidemiology and Its Role in Policy. *Advances in Nutrition* 2015; **6**(1): 5-18.
62. Aune D, Keum N, Giovannucci E, et al. Whole grain consumption and risk of cardiovascular disease, cancer, and all cause and cause specific mortality: systematic review and dose-response meta-analysis of prospective studies. *BMJ (Clinical research ed)* 2016; **353**: i2716.

63. Prospective Studies Collaboration, Whitlock G, Lewington S, et al. Body-mass index and cause-specific mortality in 900 000 adults: collaborative analyses of 57 prospective studies. *Lancet* 2009; **373**(9669): 1083-96.
64. Aune D, Lau R, Chan DSM, et al. Dairy products and colorectal cancer risk: a systematic review and meta-analysis of cohort studies. *Annals of Oncology: Official Journal of the European Society for Medical Oncology* 2012; **23**(1): 37-45.
65. Aune D, Norat T, Romundstad P, Vatten LJ. Dairy products and the risk of type 2 diabetes: a systematic review and dose-response meta-analysis of cohort studies. *The American Journal of Clinical Nutrition* 2013; **98**(4): 1066-83.
66. Guasch-Ferré M, Satija A, Blondin SA, et al. Meta-Analysis of Randomized Controlled Trials of Red Meat Consumption in Comparison With Various Comparison Diets on Cardiovascular Risk Factors. *Circulation* 2019; **139**(15): 1828-45.
67. Jayedi A, Shab-Bidar S, Eimeri S, Djafarian K. Fish consumption and risk of all-cause and cardiovascular mortality: A dose-response meta-analysis of prospective observational studies. *Public Health Nutrition* 2018; **21**(7): 1297-306.
68. Xun P, Qin B, Song Y, et al. Fish consumption and risk of stroke and its subtypes: accumulative evidence from a meta-analysis of prospective cohort studies. *European Journal of Clinical Nutrition* 2012; **66**(11): 1199-207.
69. Zhao LG, Sun JW, Yang Y, Ma X, Wang YY, Xiang YB. Fish consumption and all-cause mortality: a meta-analysis of cohort studies. *European Journal of Clinical Nutrition* 2016; **70**(2): 155-61.
70. Schwingshackl L, Knüppel S, Michels N, et al. Intake of 12 food groups and disability-adjusted life years from coronary heart disease, stroke, type 2 diabetes, and colorectal cancer in 16 European countries. *European Journal of Epidemiology* 2019; **34**(8): 765-75.
71. FAO. Food balance sheets: a handbook. Rome, Italy: Food and Agriculture Organization of the United Nations; 2001.
72. Gobbo DLC, Khatibzadeh S, Imamura F, et al. Assessing global dietary habits: a comparison of national estimates from the FAO and the Global Dietary Database. *The American Journal of Clinical Nutrition* 2015; **101**(5): 1038-46.
73. Micha R, Khatibzadeh S, Shi P, et al. Global, regional and national consumption of major food groups in 1990 and 2010: a systematic analysis including 266 country-specific nutrition surveys worldwide. *BMJ Open* 2015; **5**(9): e008705.
74. Freedman LS, Commins JM, Moler JE, et al. Pooled results from 5 validation studies of dietary self-report instruments using recovery biomarkers for energy and protein intake. *American Journal of Epidemiology* 2014; **180**(2): 172-88.
75. Rennie KL, Coward A, Jebb SA. Estimating under-reporting of energy intake in dietary surveys using an individualised method. *British Journal of Nutrition* 2007; **97**(6): 1169-76.
76. US Department of Transportation. National Household Travel Survey. 2019. https://nhts.ornl.gov/.
77. Bundesministerium für Verkehr und digitale Infrastruktur. Mobilitaet in Deutschland. 2017. http://www.mobilitaet-in-deutschland.de/MiT2017.html.
78. UK Department for Transport. National Travel Survey Statistics. 2018. https://www.gov.uk/government/collections/national-travel-survey-statistics
79. de Sá TH, Tainio M, Goodman A, et al. Health impact modelling of different travel patterns on physical activity, air pollution and road injuries for São Paulo, Brazil. *Environment international* 2017; **108**: 22-31.
80. Metropolitan Transportation Planning and Expansion Board. Search for Mobility (Pesquisa de mobilidade): Governor of the state of Sao Paulo, 2012.
81. Gong W, Yuan F, Feng G, et al. Trends in Transportation Modes and Time among Chinese Population from 2002 to 2012. *Int J Environ Res Public Health* 2020; **17**(3): 945.

82. Statistics South Africa. National Travel Survey. 2013. http://www.statssa.gov.za/publications/P0320/P03202013.pdf.
83. Ilahi A, Balać M, Axhausen KW. Existing urban transportation in Greater Jakarta: Results of agent-based modelling. *Arbeitsberichte Verkehrs-und Raumplanung* 2019; **1478**.
84. Ministry of Home Affairs. B-28 'Other Workers' By Distance From Residence To Place Of Work And Mode Of Travel To Place Of Work. India: Office of the Registrar General & Census Commissioner, India, 2011.
85. Lagos Metropolitan Area Transport Authority. Transportation and Mobility Systems in Lagos, 2015.
86. Arora A, Gadepalli R, Sharawat PK, Vaid A, A K. Low Carbon Comprehensive Mobility Plan – Visakhapatnam. Nairobi, Kenya: UNEP; 2014.
87. Kelly P, Kahlmeier S, Götschi T, et al. Systematic review and meta-analysis of reduction in all-cause mortality from walking and cycling and shape of dose response relationship. *International Journal of Behavioral Nutrition and Physical Activity* 2014; **11**(1): 132.
88. IEA. World Energy Outlook 2019. Paris: IEA, 2019.
89. IEA. World Energy Model Stated Policies Scenario. 2019. https://www.iea.org/reports/world-energy-model/stated-policies-scenario.
90. IEA. World Energy Model Sustainable Development Scenario. 2019. https://www.iea.org/reports/world-energy-model/sustainable-development-scenario.
91. WHO. The Helsinki Statement on Health in All Policies. Geneva, Switzerland: World Health Organization, 2013.
92. Burnett R, Chen H, Szyszkowicz M, et al. Global estimates of mortality associated with long-term exposure to outdoor fine particulate matter. *Proceedings of the National Academy of Sciences* 2018; **115**(38): 9592.
93. Stanaway JD, Afshin A, Gakidou E, et al. Global, regional, and national comparative risk assessment of 84 behavioural, environmental and occupational, and metabolic risks or clusters of risks for 195 countries and territories, 1990–2017: a systematic analysis for the Global Burden of Disease Study 2017. *The Lancet* 2018; **392**(10159): 1923-94.
94. Hoek G, Krishnan RM, Beelen R, et al. Long-term air pollution exposure and cardio-respiratory mortality: a review. *Environmental Health* 2013; **12**(1): 43.
95. Mozaffarian D. Dietary and Policy Priorities for Cardiovascular Disease, Diabetes, and Obesity: A Comprehensive Review. *Circulation* 2016; **133**(2): 187-225.
96. Winters M, Buehler R, Götschi T. Policies to Promote Active Travel: Evidence from Reviews of the Literature. *Current Environmental Health Reports* 2017; **4**(3): 278-85.
97. Tainio M, de Nazelle AJ, Götschi T, et al. Can air pollution negate the health benefits of cycling and walking? *Preventive Medicine* 2016; **87**: 233-6.
98. Pearce M, Strain T, Kim Y, et al. Estimating physical activity from self-reported behaviours in large-scale population studies using network harmonisation: findings from UK Biobank and associations with disease outcomes. *International Journal of Behavioral Nutrition and Physical Activity* 2020; **17**(1): 40.
99. World Bank. World Bank Indicators. 2020. https://data.worldbank.org/indicator/.
